# Two exceptionally preserved biotas from North Dakota reveal cryptic Ordovician shelf ecologies

**DOI:** 10.1073/pnas.2520246122

**Published:** 2025-11-03

**Authors:** Giovanni Mussini, Nicholas J. Butterfield

**Affiliations:** ^a^Department of Earth Sciences, University of Cambridge, Cambridge CB2 3EQ, United Kingdom

**Keywords:** exceptional preservation, Ordovician, Burgess Shale, small carbonaceous fossils, Laurentia

## Abstract

The Ordovician saw one of the greatest evolutionary radiations in the Earth’s history, precipitating the assembly of modern animal-dominated ecologies in the aftermath of the Cambrian Explosion. However, Ordovician nonmineralized faunas are rare and mostly sample ecologically marginal settings. We describe small carbonaceous fossils (SCFs), including semiarticulated elements preserving submicrometric detail, from epicratonic deposits of the Deadwood and Winnipeg formations (ND). The Osterberg SCFs, associated with biostratigraphically informative conodonts and graptolites, record two successive biotas of Cambrian-Tremadocian (the “lower Osterberg” biota) and Darriwilian (the “upper Osterberg” biota) ages, demonstrating a long-term persistence of high-quality, Burgess Shale-type microfossil preservation after the end of the Cambrian. Their components open a window on normal marine, well-oxygenated Ordovician shelf habitats, revealing taxa and functional morphologies unrecorded by coeval macrofossils. These include specialized grazing and predatory molluskan radulae, triturative crustaceomorph molars, the oldest known eurypterid-type cuticles, and microphagous priapulid worms. The Osterberg fossils attest to an Ordovician co-occurrence of cryptic taxa and feeding adaptations, reminiscent of the most ecologically modern Cambrian biotas, alongside classic later-Paleozoic forms like colonial zooplankton and biomineralized early vertebrates. By contrast, they do not record classic Burgess Shale-style taxa typical of marginal or deeper-water Ordovician assemblages. These results demonstrate a lasting presence of cryptic, modern-style shelf faunas throughout the earliest Paleozoic, suggesting that exceptional Ordovician macrofossil sites are unrepresentative of the broader state of their coeval biosphere.

## Significance

The Ordovician is one of the most important periods in Earth history, witnessing the elaboration of modern-style ecosystems after the “Cambrian Explosion” of animals. However, exceptional fossils from this period are mostly confined to marginal environments, obscuring the most widespread and habitable ecosystems of their day. We describe Ordovician exceptional fossils from shallow-water, well-oxygenated habitats, representative of the widespread continental seas of the time. These fossils suggest that classic Ordovician exceptional biotas record relative evolutionary “backwaters”: The rest of the biosphere may have been dominated by extinct skeletonized taxa and cryptic, modern-style animals.

The Ordovician Period records one of the most extensive Phanerozoic evolutionary radiations (1–3). It saw the rise to ecological dominance of extant animal orders and classes alongside life modes that define modern biotas, including multitiered suspension feeders, a diverse zooplankton, and large nektonic predators (1, 4). However, the tempo and mode of Ordovician biodiversification remain debated, largely due to a discontinuous global faunal record in which a few skeletonized groups remain disproportionately represented (5).

Exceptional preservation deposits—“Konservat-Lagerstätten” (6)—chronicle the evolution of the nonbiomineralized faunas dominating early Paleozoic communities (6, 7). In the Cambrian, they are epitomized by Burgess Shale-type (BST) deposits, preserving carcasses primarily as carbonaceous compressions (8). However, Ordovician Konservat-Lagerstätten are rarer than Cambrian counterparts and typically show lower-fidelity preservation, often heavily biased toward substantially sclerotized or biomineralized parts already by the Tremadocian (9–11): This post-Cambrian degradation of exceptional preservation has been linked to declines in favorable outer detrital environments, escalating burrowing and carcass consumption, and secular changes in ocean chemistry (7, 12). Moreover, most Ordovician Konservat-Lagerstätten record high-latitude, refugial, or marginal marine settings; even open-shelf examples occupied relatively low-bioturbation, dysoxic deeper-water paleoenvironments (9–11, 13).

In the Cambrian, the paleoenvironmental window of BST preservation is increasingly being expanded by small carbonaceous fossils (SCFs): BST micro- to mesofossils more robust to reworking, bioturbation, and ingestion than macroscopic counterparts (14). Cambrian SCFs have captured exceptionally preserved metazoans beyond the marginal, freshwater-influenced, or outer detrital settings associated with BST Konservat-Lagerstätten—and into the well-oxygenated shelf habitats hosting most Phanerozoic marine biodiversity (15, 16). By contrast, Ordovician SCFs are rare (13, 17, 18) and record taxonomically restricted metazoans (mainly arthropod and scalidophoran fragments) (18, 19), rather than diverse assemblages yielding more representative views of original paleocommunities (7, 8).

We describe diverse Ordovician SCFs from the subsurface of the Deadwood and Winnipeg formations of North Dakota, associated with biostratigraphically informative conodonts and graptolites. These fossils capture disparate latest Cambrian to Darriwilian nonmineralized faunas, revealing life in well-aerated, normal marine epeiric habitats during a critical phase of Paleozoic biodiversification.

## Results

### Geological Context.

Studied fossils were extracted from two closely placed, lithologically comparable drillholes of the Osterberg 21-2 (48.802903 N, -101.642301 W) and Osterberg 22X (48.802904 N, -101.642302 W) oil exploration wells in Renville County, North Dakota, situated within the Williston Basin—a major intracratonic embayment spanning much of the Paleozoic (20, 21). The Osterberg drillcores span two Cambrian–Ordovician stratigraphic units separated by a first-order erosional unconformity (22–24): the late Cambrian—early Ordovician Deadwood Formation, deposited during the latter part of the Neoproterozoic to mid-Ordovician Sauk transgression, and the middle—late Ordovician Winnipeg Formation, deposited during the mid-Ordovician through early Devonian Tippecanoe transgression (*SI Appendix*, Fig. S1).

The Deadwood Formation extends across the eastern Western Canada Sedimentary Basin in Saskatchewan and Alberta, into the Williston Basin of eastern Montana, southern Saskatchewan, south-western Manitoba, and North Dakota, and outcrops in the Black Hills of South Dakota—its type locality (23, 25). It records siliciclastic, carbonate and evaporite packages resting uncomformably on crystalline basement rocks (23). The Deadwood Formation underlies the major erosional unconformity separating the Sauk megasequence from overlying Tippecanoe-aged strata (23, 26). In the Williston Basin, it comprises six informal units (A-F in ascending order), correlatable among subsurface cores from the Dakotas, Montana, and Saskatchewan based on their lithology and wireline logs (23, 27).

**Fig. 1.**
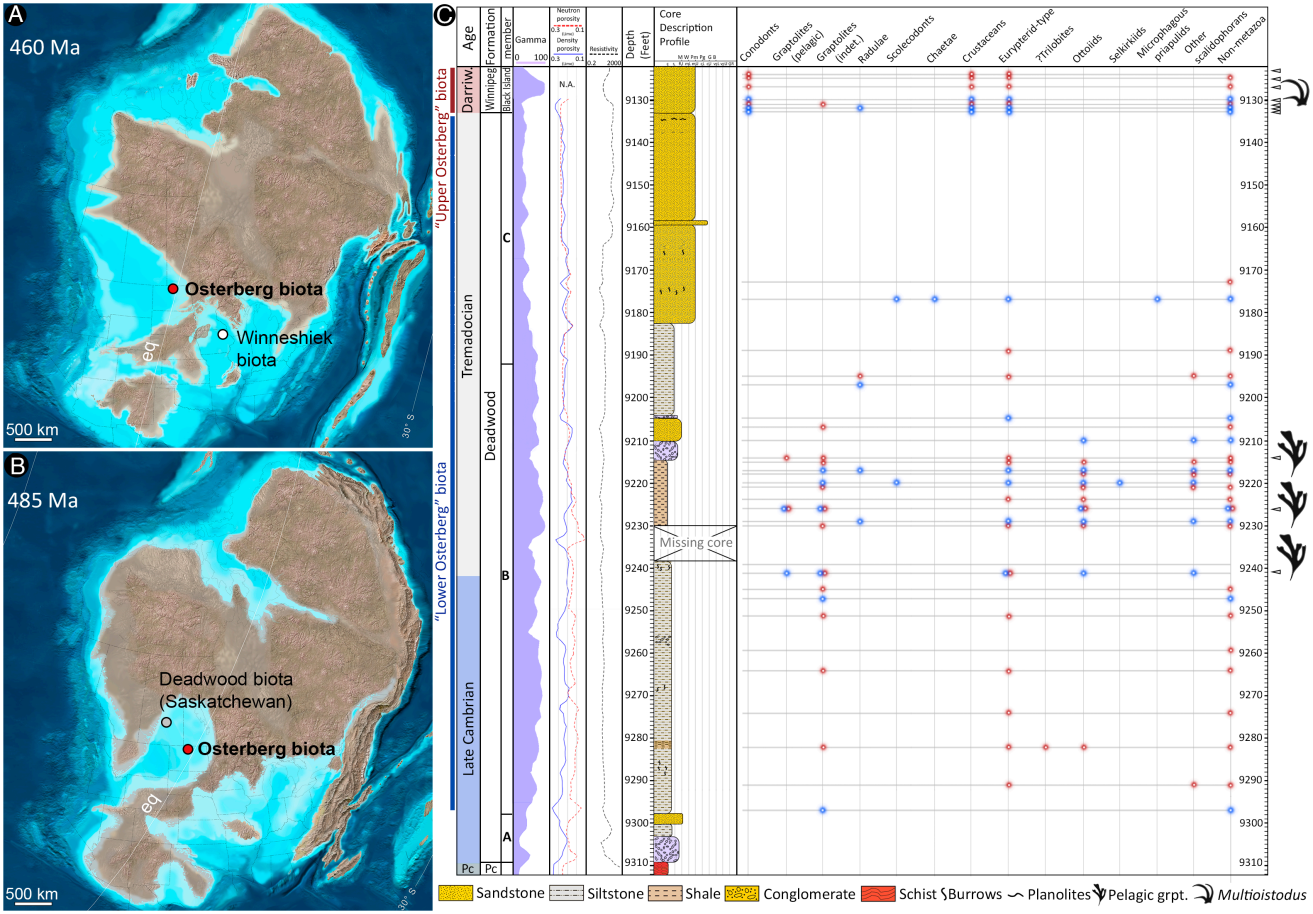
Paleogeographical position and stratigraphy of the Osterberg biotas. (*A*) Paleoenvironmental map of North America (Laurentia) during the middle Ordovician (c. 460 Ma), corresponding to the time of deposition of the upper Osterberg biota (Winnipeg Formation). Approximate locations of the Osterberg biotas and the broadly coeval Winneshiek biota shown by colored dots. (*B*) Paleoenvironmental map of Laurentia at approximately the Cambrian–Ordovician boundary (c. 485 Ma), corresponding to the oldest time of deposition of the lower Osterberg biota (Deadwood Formation). Locations of the Osterberg biota and the broadly coeval Deadwood biota of Saskatchewan shown by colored dots. (*C*) Diagrammatic representation of predominant lithologies recorded by the Osterberg drillcores, with corresponding age estimates, drillcore depths, and gamma, resistivity, and porosity logs after the present study and (27, 30). SCF occurrences at each sampled depth (horizontal lines) are indicated by red (for the Osterberg 22X drillcore) and blue dots (for the Osterberg 22N-2 drillcore) in the corresponding vertical columns. grpt, graptolites. Source maps © 2023 Colorado Plateau Geosystems Inc.

Members A–F record marginal marine deposits (the conglomerate/sandstone-dominated member A) succeeded by well-aerated shallow marine (the glauconite, siltstone, and sandstone-dominated member B) to nearshore progradational deposits (members C–F, dominated by sandstone, carbonate, siliciclastic mudstone, and siltstone packages) (20, 24, 27). In North Dakota, the Deadwood Formation thins to the east (cratonward), only recording members A-C in the Osterberg drillcores (27) (Fig. 1*C*). Members A–F are dated as Furongian to Floian based on skeletal fossils from other subsurface cores across Montana and North Dakota (20, 21, 28, 29). Member A is typically unfossiliferous (23). Member B hosts dendroid graptolites and rarer trilobites; the Cambrian (Furongian)—Ordovician (Tremadocian) boundary is placed within this member, at the lowermost occurrences of *Symphysurina* and/or *Euloma* trilobites (20, 23). Members C–D, F yield trilobites, gastropods, brachiopods, and eocrinoids, correlatable with regional Tremadocian (members C-D) and Floian (member F) shelly faunas (20, 23, 28, 29). These occurrences of diverse shelly taxa, particularly trilobites, graptolites, and crinoids, support predominant well-aerated, normal marine conditions in members B through F (20).

In the Osterberg drillcores, Deadwood member C uncomformably underlies the middle–late Ordovician Winnipeg Formation, deposited during the earliest part of the Tippecanoe transgression (Fig. 1*C*) (22, 23). The Winnipeg Formation extends across Saskatchewan, southwestern Manitoba, Minnesota, North and South Dakota, Wyoming, and Montana; like the Deadwood Formation, it is mostly encountered in the subsurface (24, 26, 31). It represents a transgressive sequence of nearshore—lower-offshore settings recorded (from oldest to youngest) by sandstone/siltstone with shaly interbeds (Black Island Member); green-gray shale (Icebox Member); and limestone (Roughlock Member) (22, 24, 26, 31, 32) packages.

Late Ordovician (Sandbian) conodonts occur in outcrops of the Icebox and Roughlock members of the Winnipeg Formation in the northern Black Hills (31). Similar faunas characterize the Icebox—Roughlock members in North Dakotan drillcores [(26), figure 9] and probable time-equivalent units in Manitoba (31). Significantly, middle Ordovician (Darriwilian) conodonts were recovered from both Osterberg drillcores at depths of 9,124 to 9,133 feet (~2,781.1 to 2,783.7 m; Fig. 1*C*), identifying their host strata of sandstone/siltstone with shaly interbeds as part of the lowermost Winnipeg (Black Island) member lying immediately above Deadwood strata (see *The Osterberg Biotas*, Conodonts). Therefore, the Osterberg samples collectively span two first-order transgressive megasequences (Sauk and Tippecanoe) framing the early Phanerozoic history of Laurentia.

### The Osterberg Biotas.

The Osterberg samples yielded 4,116 micro- to mesofossils, including 2,181 recognizable metazoan specimens for which we provide a general account below. Co-occurring taxonomically indeterminate specimens and nonmetazoans are presented in our *SI Appendix*, Figs. S2–S5. We collectively refer to fossils from Winnipeg Formation horizons (9,124 to 9,133 feet of depth), deposited during the Tippecanoe transgression, as the “upper Osterberg” biota; and to Deadwood Formation specimens, dating to the time of the Sauk transgression (9,173 to 9,297 feet of depth), as the “lower Osterberg” biota (Fig. 1*C*).

#### Conodonts.

**Fig. 2.**
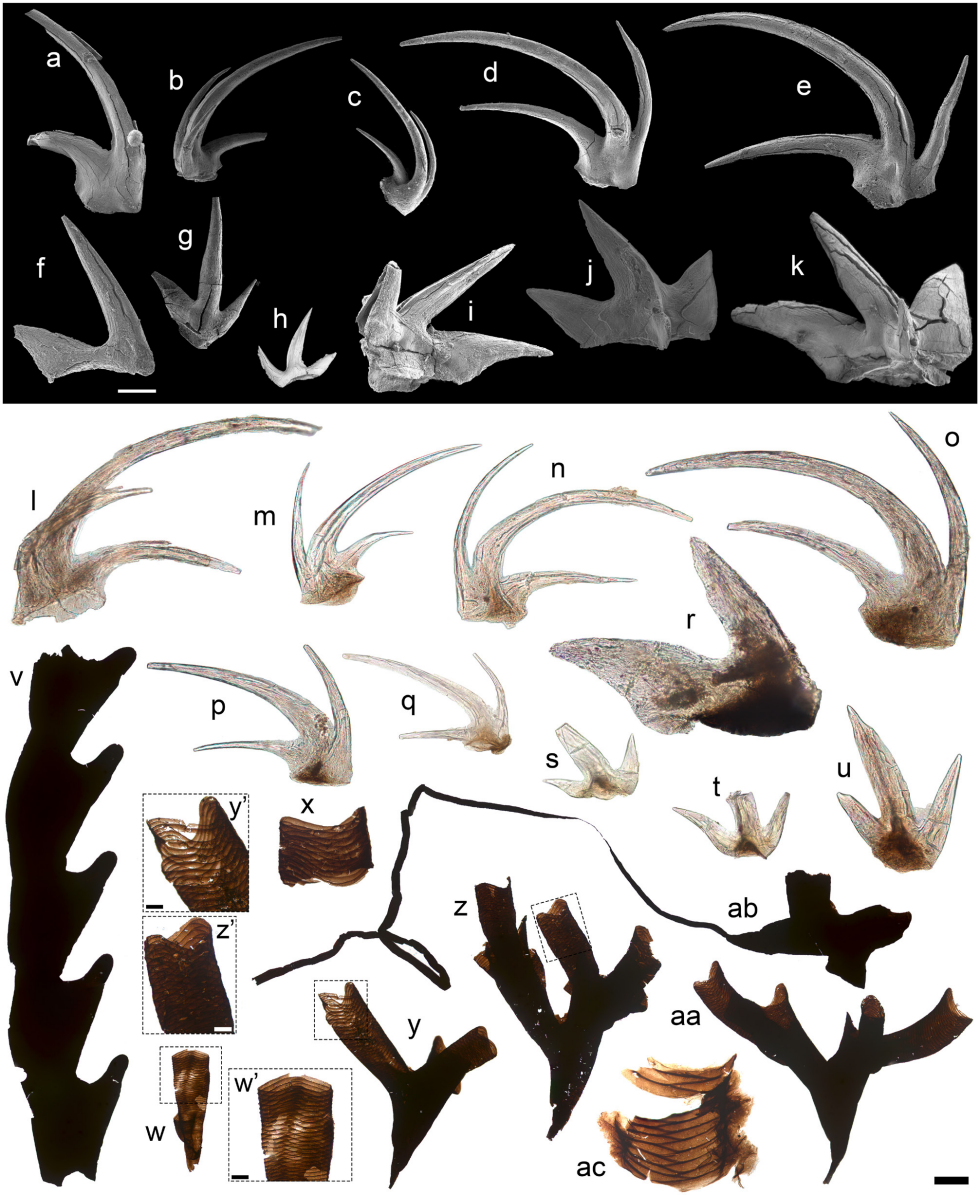
Conodonts from the upper Osterberg biota (a–u) and pterobranchs from the lower Osterberg biota (v–ac). a–e, *Multioistodus subdentatus*, SEM imaging. a, Sc element; b, Sa element (note posteriorly broken and detached cusp margin); c, P element; d and e, Sb elements. f–k, cf.*Neomultioistodus? clypeus*, SEM imaging. f–h, transition series of ramiform elements; i–k, pastinate P elements. l–p, *Multioistodus subdentatus*, optical imaging. l, Sa element; m–o, Sb elements; p and q, P elements.r–u, cf.*Neomultioistodus? lypeus*. r, pastinate element; s–u, transition series of ramiform elements. v–ac, pterobranchs from the Osterberg biota. v, large fragment of tubaria expressing uniseriate lateral thecae. w, tapering basal portion of graptolite tube showing suture details (w’). x, ac, tubular fragments showing zigzagging sutures. y-aa, branching basal portions of graptolite tubaria with tapering siculae, with details of sutures shown in y’ and z’. ab, specimen showing tapering sicula continuous with nema. Scale: 100 µm except for b and c (200 µm). Slide numbers and England Finder coordinates given in Dataset S1.

Conodonts, a group of Cambrian to Triassic chordates with bioapatite teeth, are outstanding biostratigraphic indicators due to their diversity, abundance, and preservation potential (33). Despite the small volumes of available drillcore samples, the upper Osterberg biota yielded disparate ramiform (branching), coniform (cone-like), and pectiniform (comb-like) teeth, including a coherent plexus of middle Ordovician genera recorded by incomplete element series (*SI Appendix*, Fig. S3 and *Supporting Information Text*). Most specimens show organic, dark brown basal bodies together with bioapatite crowns (Fig. 2a–u and *SI Appendix*, Fig. S3).

The most numerous (N = 266) conodont in the upper Osterberg biota is the Darriwilian *Multioistodus*, showing a diagnostic falciform, recurved carinate cusp flanked by prominent denticles developing from its base. The Osterberg specimens are attributed to the type species *M. subdentatus* based on its four-member apparatus of alate Sa [Fig. 2b and l cf. (34), pl. 3 figure 3], bipennate Sb [Fig. 2d, e, n, and o cf. (35), figure 4.11; (34) pl. 3 figure 2], pastinate [Fig. 2c, p, and q cf. (35), figure 4. 12; (34), pl. 3 figure 7] or digyrate [Fig. 2m cf. (34), pl. 3 figure 4] P elements, and dolabrate Sc elements [Fig. 2a cf. (34–37)]. *M. subdentatus* has been reported from Missouri, Oklahoma, Utah, and the Winneshiek shale of Iowa (17, 34–38): All its occurrences are Darriwilian. The co-occurring apparatus of the coeval (Darriwilian) and morphologically similar *Neomultioistodus? clypeus* (34, 39) (N = 33) differs by having pastinate teeth with broad, flattened lateral denticles (Fig. 2i–k and r), also associated with a transition series of ramiform elements (Fig. 2f–h and s–u) (34, 39).

#### Pterobranchs.

Tubes from graptolite pterobranchs, consisting of collagenous periderms with characteristic zigzag sutures (Fig. 2 x, y’, z’, ac) (40, 41), were recovered from the lower Osterberg biota (Fig. 1*C*). The Osterberg graptolites include specimens preserving branching stacks (“stipes”), up to ~2.5 mm long and comprising uniseriate lateral thecae (e.g., Fig. 2v and *SI Appendix*, Fig. S4 o and p). Some specimens preserve a filiform nema (thin tubular extension; Fig. 2ab) borne on a tapering tube or “sicula” (42) from which the stipes branch out in a conical pattern following apparent bi- to quadradiate configurations (Fig. 2 y–aa and *SI Appendix*, Fig. S4 c, d, and f). The nema, reflecting attachment to floating debris (42), identifies these SCFs as pelagic graptolites—only known from Ordovician and younger deposits and typifying normal marine planktonic assemblages (42, 43). The flattened, fragmentary nature of the material hinders recognition of diagnostic colony shapes; however, the combination of a nema-bearing sicula, conical shape, and apparent bi- to quadriradiate morphologies suggests attribution to the Tremadocian *Rhabdinopora* (20, 42, 44).

#### Mollusks.

**Fig. 3.**
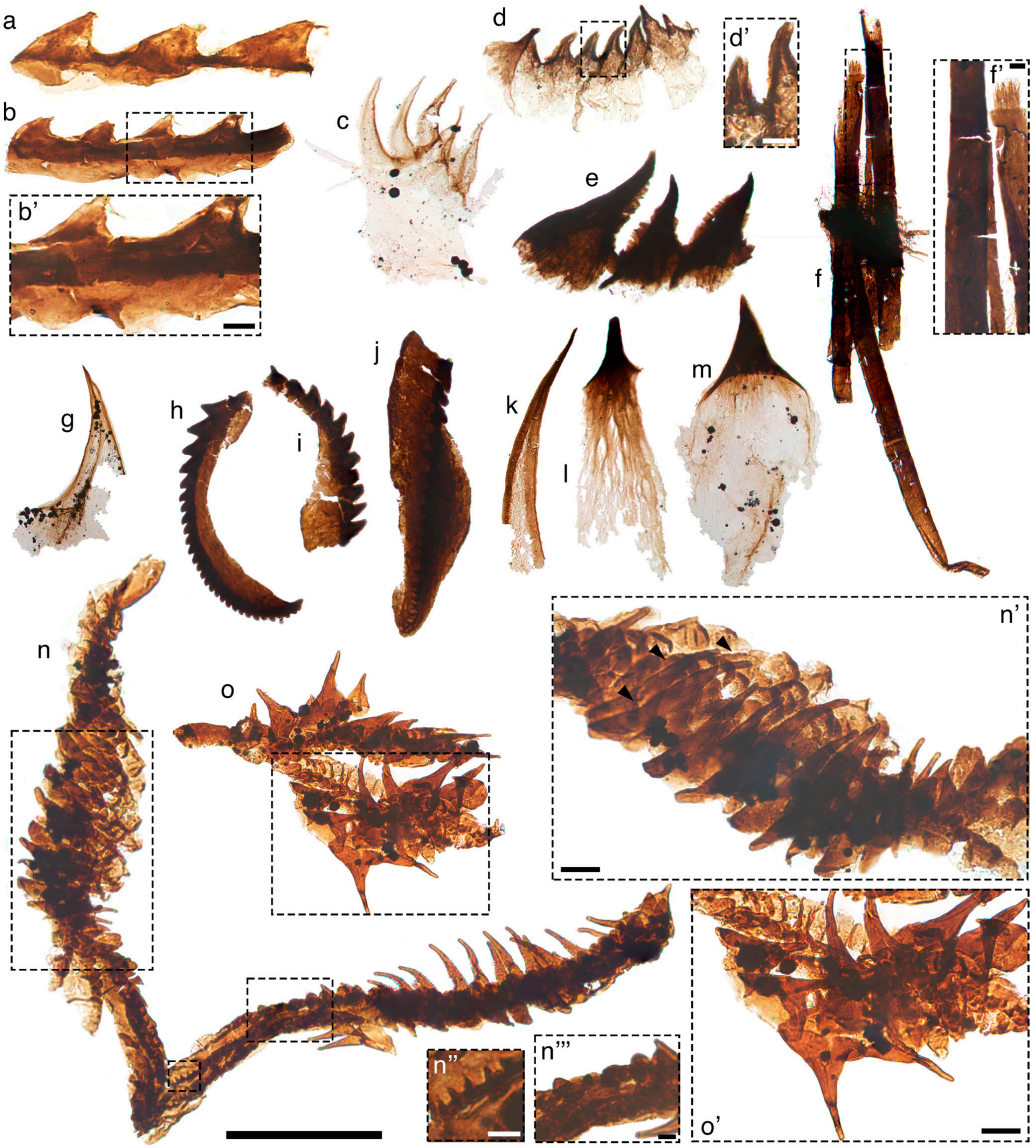
Lophotrochozoans from the upper (a and b) and lower (c–o) Osterberg biotas. a and b, fragments of polystichous radulae; teeth shown in b’. c putative uniseriate radula with recumbent teeth; note probable obliquely compressed basal membrane. d, uniseriate radulae with recumbent teeth; teeth shown in d’, highlighting blunt tips with possible terminal wear. e, sclerotized fragment of uniseriate radula. f, bundle of annelid-type chaetae; barred construction and brush-like termination shown in f’. g, isolated tooth from probable uniseriate radula (cf. c). h–j, scolecodonts. k, isolated tooth from probable uniseriate radula (cf. c and d). l and m, isolated peg-like to spinose teeth from radular apparatuses. n, semiarticulated polystichous radula bearing hook-like teeth; three lateral hook series (arrowheads) shown in n’; median teeth in n’’; and flanking tubercle-like teeth in n’’’. o, section of semiarticulated polystichous radula bearing hook-like teeth; hook-like teeth flanking tuberculate elements shown in o’. Scale: 50 µm except for a (25 µm), b’, f’, n’-n’’’, o’ (5 µm), f (200 µm), g–j, k (100 µm), n (75 µm), o (40 µm) Slide numbers and England Finder coordinates given in Dataset S1.

**Fig. 4.**
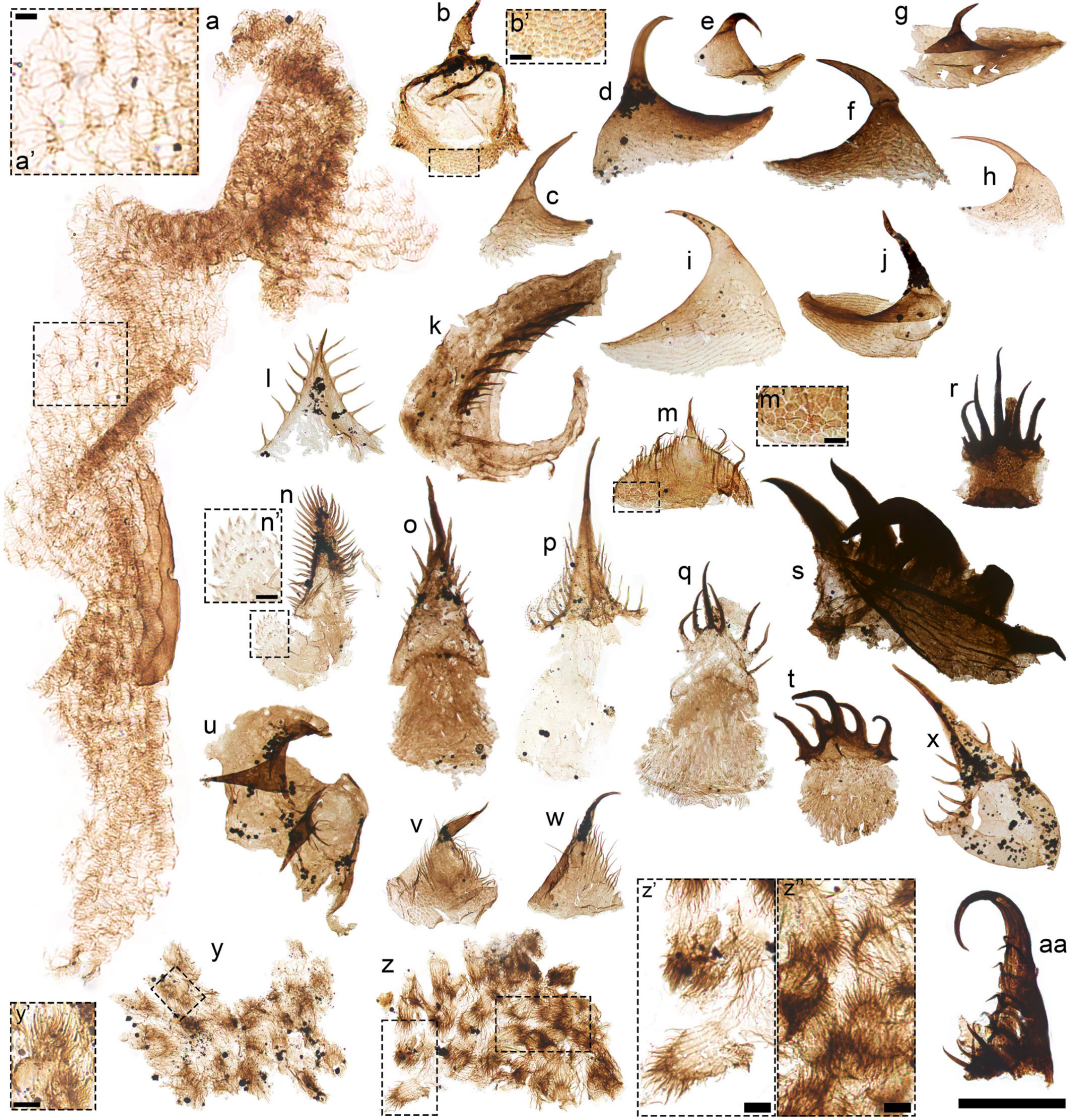
Scalidophorans from the lower Osterberg biota. a, semiarticulated putative trunk scalids; detail shown in a’. b, spine with flaring base adorned by cuticular scales, shown in b’. c–j, falcate hooks with ‘shoe-shaped’ base. k, *Ottoia*-type introvert hook. l, *Selkirkia*-type tooth. m, v, w, ottoiid Type D tooth (62); basal pad ornamentation shown in m’. n, ottoiid tooth intermediate between types C and D (62); basal pad ornamentation shown in n’. o, ottoiid Type C tooth (62). p, Ottoiid tooth intermediate between types B and C (62). q-t, ottoiid Type B teeth (62). u, flaring spines recording possible trunk sclerites. x, aa, priapulomorph introvert hooks. y-z, semiarticulated teeth from microphagous priapulids; sclerites shown in y’ and z’–z’’. Scale: 100 µm except for a (40 µm), a’ (4 µm), b’, n’, m’, y’, z’–z’’ (10 µm). Slide numbers and England Finder coordinates given in Dataset S1.

**Fig. 5.**
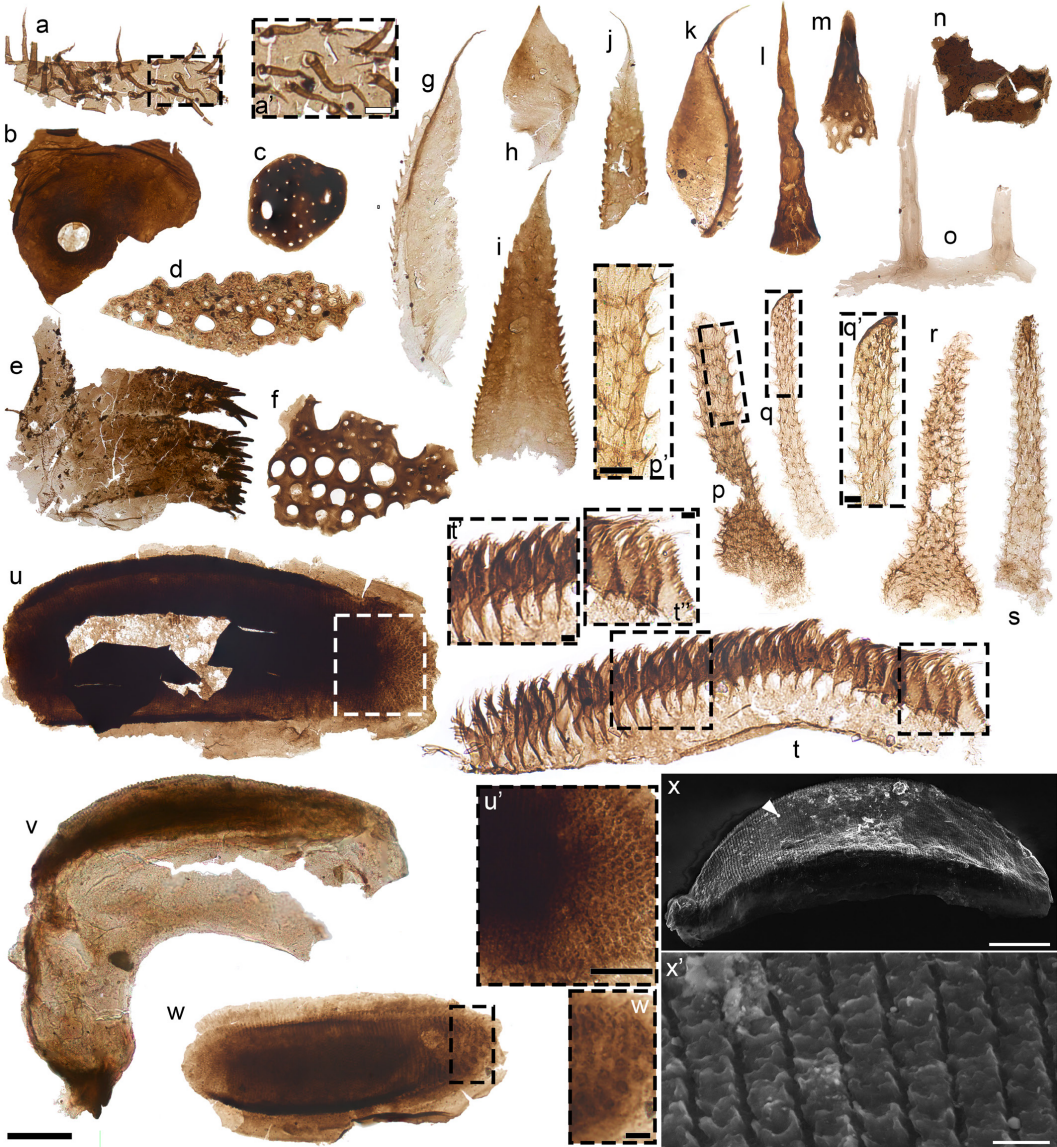
Arthropods from the lower (a, b, e, g–l, n, o, and t) and upper (c, d, f, m, p–s, and u–x) Osterberg biotas. a, setose eurypterid-type cuticle; setal pores shown in a’. b, cuticle with lacuna and plicae. c and d, eurypterid-type cuticles with ellipsoid/rounded openings. f, cuticle with rounded lacunae. g–k, cuticular “paddles.” l, o, unornamented spine [cf. (68, 69)]. m, perforated spine. n, cuticle fragment with lacunae. p–s, eurypterid-type respiratory organs; polygonally arranged protrusions shown in p’, q’. t, uniseriate “feathery” plates; bases and filamentous edges shown in t’–t’’. u–w, molars photographed under optical microscopy; tubercles shown in u’ and w’; note cuticular lobe in v. x, molar photographed under SEM, showing scaly lineations (x’). Scale: 100 µm except for n (200 µm), t (10 µm), t’–t’’ (1 µm), u’ (50 µm), w’, p’, q’ (10 µm), x’ (5 µm). Slide numbers and England Finder coordinates given in Dataset S1.

The upper and lower Osterberg SCFs collectively include 14 specimens of radulae—chitinous, toothed molluskan mouthparts. In lower Osterberg samples, 45 to 70-µm-long, semiarticulated uniseriate apparati with basally abutting, gently recumbent teeth with bilayered construction (Fig. 3d, cf. c; N = 5) find counterparts among similarly uniseriate radulae from the Stage 4 File Haidar Formation of Baltica [(45), figure 3 L–O, T, and U] and extant sacoglossan gastropods (46, 47). Isolated spines with flaring fibrous bases (Fig. 3 l and m; N = 2) and slender blade-like elements with thickened margins (Fig. 3 k and g; N = 2), up to ~80 µm long, are also comparable to File Haidar specimens [(45), figure 3 AA, AF, P, Q, and X] and sacoglossan forms [(47), figure 17]. In the upper Osterberg biota, 50 to 95 µm-long carbonaceous straps fringed by regularly spaced, gently recumbent semiequilateral teeth (Fig. 3 a and b) record probable teeth columns from compound (polystichous) radulae for substrate rasping or scraping (45) (N = 3). Counterparts include early Cambrian SCFs from Baltica [(45), figure 3 A–G] and extant gastropod radulae [(48), figures 4 and 5A].

Two complexes of laterally differentiated parallel tooth chains (Fig. 3 n–o) were recovered in the lower Osterberg biota (Dataset S1). On each margin, these polystichous elements bear three parallel rows of slender, semilunate “hooks” with flaring bases (Fig. 3 n’, o’). Of these, the most external row comprises ~20 to 25-µm-long hooks; the more medial consists of hooks ~12 to 15 µm long. The most extensively articulated specimen shows minute (~2 to 4 µm long) uniseriate cuspidate elements medially (Fig. 3n’’). Flanking them, but medial to the lateral series of hook-like elements, are two parallel rows of ~5- to 8-µm-long, tubercle-like semiequilateral teeth (Fig. 3n’’’).

Multiseriate rows of hook-like teeth consistently associate with predatory habits in extant mollusks. Close counterparts occur in muricid [(49), figures 16, 27-29; (50), figures 4f and 5c] and tonnoidean gastropods [(51), figure 2]. Among cephalopods, the Osterberg specimens differ from nautilid radulae in their lower number of parallel element series (>10 in nautilids) and absence of robust, flattened marginal plates [(52), figure 2; (53), figure 11.3]. However, their slender falcate hooks, lack of marginal plates, and fewer parallel element series, with a median row of minute rhachidian teeth, compare favorably with those of Paleozoic orthoceratid nautiloids [e.g., (53), figure 11.4a; (52), figure 1], suggesting a cephalopod affiliation consistent with normal marine origins for the Osterberg faunas. By contrast, gastropods with comparable radulae are understood to have Mesozoic origins (54, 55).

#### Annelids.

The lower Osterberg biota yielded rare (N = 3) scolecodonts—the chitinous jaws of eunicid annelids. These fragmentary, ~110 to 200-µm-long SCFs are identified by their optically dense, serrated subtriangular teeth arranged into a single series borne on a blade-like chitinous ridge (Fig. 3 h–j). An annelid producer is also consistent with bundled straight-sided, ~240 to 500-µm-long strap-shaped chaetae, showing a characteristic lophotrochozoan microstructure of longitudinal parallel fibers (56) and a brush-like tip (Fig. 3f). These chaetae consist of segments separated by regular bands of lighter cuticle, with variable degrees of preservation (Fig. 5f’). Bundled, multiarticulated chaetae occur in diverse living annelid families (57, 58). Brush-like tips also find counterparts among both extant polychaetes, including eunicids (57), and early Cambrian SCFs from Baltica (58, 59).

#### Scalidophorans.

Scalidophorans are vermiform ecdysozoans comprising priapulids, loriciferans, and kinorhynchs (60). Priapulid SCFs were recovered in the lower Osterberg biota, where they include spinose and hook-like sclerites likely from the introvert and trunk regions (Fig. 4 b–j, u, and aa and *SI Appendix*, *Supporting Information Text*) and more diagnostic pharyngeal teeth (N = 87; Fig. 4 l–t, v, and w): The latter are identified by sclerotized, denticulate marginal arches, apical prongs, basal spurs, and/or basal pads of delicate smooth or scaly (Fig. 4 m’ and n’) cuticle (61, 62). U-shaped, spinulose teeth (N = 86) match *Ottoia*-type forms from the Deadwood Formation of Saskatchewan (19, 62). A 140-μm-long cuspidate element with well-spaced spinose denticles (Fig. 4l) matches “type A” teeth in the Burgess Shale priapulid *Selkirkia* (62, 63).

The lower Osterberg priapulomorphs also include 4 semiarticulated clusters of 30 to 40-μm-long, poorly sclerotized comb-shaped sclerites bearing densely spaced, 5 to 10-μm-long filiform denticles (Fig. 4 y, z, y’, z’-z’’). These SCFs are morphologically comparable to the U-shaped, bristled brush-like teeth of extant microphagous priapulids (Tubiluchidae and Meiopriapulidae), suggesting similar benthic microdetritivory (64); a similar particle-feeding morphotype characterizes the Cambrian priapulid *Kraytdraco* from the Wuliuan Bright Angel Formation (AZ) (16). A ~360-μm-long semiarticulated cluster (Fig. 4a) comprises similarly comb-like, filament-bearing elements. However, these are smaller (4 to 6 μm long) and optically lighter than co-occurring teeth and lack a diagnostic arch, prong, or spur: As such, these sclerites are more closely comparable to pectinate locomotory trunk scalids [(65), figure 3C].

#### Crustaceomorphs.

274 molar surfaces—apical grinding portions of arthropod mandibular complexes (66)—were recovered in all studied samples from the upper Osterberg biota. These ~350 to 600-μm-long and ~110 to 150-μm-wide SCFs show arcuate profiles and ovoid apical surfaces, with densely spaced, transverse denticulate (“scaly”) rows spanning the entire molar width (Fig. 5 u–x and *SI Appendix*, Fig. S5 a–d). The constituent scales are conspicuously three-dimensional. Each has a square cross-section and a depressed apical ‘socket’ with cusps protruding from its corners (Fig. 5x’). At one end of the molar’s longitudinal axis, scaly lineations grade into a cuticular pad adorned by sparser, rounded tubercles (Fig. 5 u’ and w’ and *SI Appendix*, Fig. S5b’). The molar surfaces occasionally articulate with broad cuticular flanges which follow their same arching trajectory (Fig. 5v), denoting original continuity with a mandibular lobe (66, 67).

**Fig. 6.**
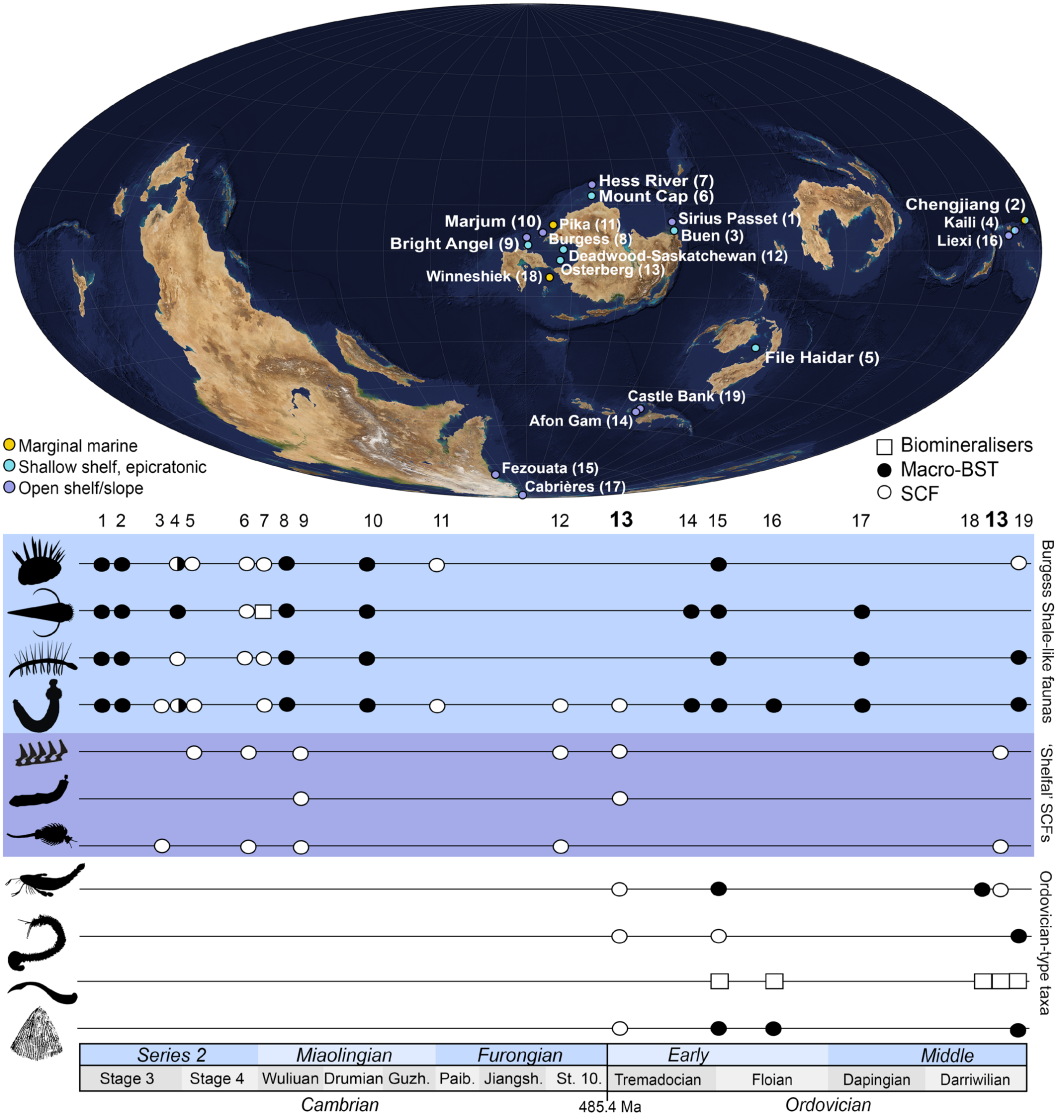
Paleoecological coverage and faunal composition of Cambrian–Ordovician exceptional biotas. Map of representative early Cambrian to middle Ordovician Fossil-Lagerstätten; continental positions correspond to a Darriwilian age (c. 460 Ma). Locations on the map are color-coded according to their depositional context. Numbers in brackets for each deposit are associated with their approximate age in the timeline chart. Black silhouettes denote, from *Top* to *Bottom*: wiwaxiids, hyoliths, lobopodians, macrophagous priapulomorphs, belt-like radulae, microphagous priapulomorphs, particle-grinding crustaceomorphs, eurypterids, eunicids, euconodonts, and pelagic graptolites. Data from ref. 10, 11, 13, 16, 17, 45, 58, 63, 82, and 86–94. Silhouettes except for radula (reproduced under a Creative Common Open Access licence from ref. 46) from phylopic.com and reproduced under the following licences: https://creativecommons.org/publicdomain/zero/1.0/ (*Wiwaxia*); https://creativecommons.org/licenses/by/4.0/ (hyolith; Martin Smith); https://creativecommons.org/licenses/by-sa/3.0/ (*Hallucigenia*; Caleb M. Brown); https://creativecommons.org/publicdomain/zero/1.0/ (priapulid); (https://creativecommons.org/publicdomain/mark/1.0/ (branchiopod); https://creativecommons.org/licenses/by/4.0/ (*Meiopriapulus*; Joel Vikberg Wernström); https://creativecommons.org/licenses/by/3.0/ (eurypterid; drawing by Dmitry Bogdanov, vectorized by Roberto Díaz Sibaja); https://creativecommons.org/licenses/by/4.0/ (eunicid; Smithsonian Environmental Research Center and T. Michael Keesey); https://creativecommons.org/licenses/by/3.0/ (euconodont; Jaime Headden); https://creativecommons.org/licenses/by/3.0/ (graptolite; credit, DW Bapst, modified from Cooper et al., 1998). Map credit: Carl-August Wingårdh.

Among mandibulate arthropods (66), branchiopod molars are characterized by elongate outlines and many closely spaced scaly lineations (67, 70). However, the upper Osterberg specimens lack the diagnostic asymmetrical variations in scale size and morphology, setose fringes, and oblique spinose fields of branchiopod molars, including Cambrian SCF examples (67, 71). Ovoid molars with finely lineated scaly rows also characterize some eumalacostracans (Syncarida) which, unlike branchiopod examples (but like the Osterberg SCFs), express a posterior tuberculate area [(66), figure 6E]. Given evidence for Devonian origins of eumalacostracans (72), the Osterberg molars may record plesiomorphic, syncarid-like malacostracans predating the divergence of Eumalacostraca (72, 73). By analogy with living crustaceans, the robust denticulate scales of the Osterberg SCFs suggest grinding capabilities, associated with the consumption of relatively recalcitrant debris or phytoplankton (66, 67, 70, 74).

#### Other arthropods.

205 cuticular fragments extracted from 24 horizons spanning the upper and lower Osterberg biotas (Fig. 1*C*) show mutually coherent morphologies characteristic of eurypterid chelicerates, previously described from Darriwilian to Permian deposits (75). These include distinctive perforated cuticles (Fig. 5a) supporting flexible, hollow setae with basal pores [cf. (68), pl. 2 figure 25, 27; (75), figure 4 b and c; (76), figure 9E; (77), pl. IV figure 13]; smooth cuticles with rounded to ellipsoid openings [Fig. 5 c and d cf. (78), figure 9K; (76), figure 9B]; large, raised pore-like lacunae [Fig. 5 f and n and *SI Appendix*, Fig. S5g cf. (77), pl. IV, figures 7 and 8], occasionally associated with bordering plicae [Fig. 5b cf. (68), pl. I figure 6]; cuticles bearing irregularly distributed ellipsoid tubercles [*SI Appendix*, Fig. S5e cf. (75), figure 23]; cuticles with irregular micron-scale punctae [*SI Appendix*, Fig. S5o cf. (68), figure 5]; and erect “tube-like” (69) hollow spines, occasionally emerging from a continuous base [Fig. 5o and *SI Appendix*, Fig. S5n cf. (69), pl. 6-8].

Characteristic eurypterid-type SCFs recovered from upper Osterberg samples also include 200 to 300-μm-long hollow, subconical extensions of delicate cuticle (N = 21) with flaring bases. These specimens extend apically into rounded, optically denser (i.e., likely sclerotized) cuticular “caps” (Fig. 5 p–s and *SI Appendix*, Fig. S5 i and j). Below the cap, the surface of each specimens expresses a polygonal meshwork of ridges. The nodes of this meshwork extend into triradial, columnar cuticular projections with flattened tips, standing proud of the surface by 6 to 10 μm (Fig. 5 p’, q’). Equivalent columnar projections arranged in meshwork-like patterns distinguish the diagnostic tubular to subconical respiratory organs (*Kiemenplatten*) of eurypterids (76–79). The blunt, rounded cuticular cap of the Osterberg examples contrasts with the larger, pointed apical cones of some geologically younger *Kiemenplatten* (79). These differences are unlikely to be merely taphonomic given the smooth, nonfrayed margins, and lack of apparent folding observed in the caps of the Osterberg specimens (Fig. 5q’ and *SI Appendix*, Fig. S5j): These features argue against postmortem deformation or breakage of originally more extensive sclerotized spines. However, apical projections differ widely (and can be absent altogether) among eurypterids (76–78). As inferred for *Kiemenplatten,* the Osterberg specimens are hollow, suggesting gas-exchange functions (79).

Also consistent with eurypterids are 130 to 260-μm-long cuticular “paddles” from the lower Osterberg samples. These SCFs (N = 17) include elongate isosceles triangular elements with uniseriate, distally pointing spinules on each long side (Fig. 5i) and arcuate, asymmetrical forms with a recurved apical projection and spinules only on their convex margin, lateral to a darker cuticular band (Fig. 5 g–k and *SI Appendix*, Fig. S5 k–m). Similar arcuate, marginally serrated structures, showing a darker submarginal band and recurrently associated with eurypterid cuticles [(76), figure 7 D and E], have been reported from the Devonian of Poland (76) and the Silurian of North Africa (68), and interpreted as part of eurypterid gnathobases (76) or other spinose appendages (68). By contrast, the triangular elements are more comparable to eurypterid fixed spines with lateral serrations [(75), figure 8B].

These eurypterid-like SCFs co-occur with diverse fragments potentially attributable to other arthropods, including finely perforated cuticles and unornamented spines (*SI Appendix*, Fig. S5 f and p), probable gnathobasic edges (Fig. 5e and *SI Appendix*, *Supporting Information Text*) and a series of subtriangular, laterally asymmetrical and basally abutting “feathery” plates (Fig. 5t) comparable to respiratory organs from trilobite upper limb branches (80) (*SI Appendix*, *Supporting Information Text*).

## Discussion

The Osterberg biotas record micro- and mesofossil BST preservation in normal marine Ordovician epeiric environments (14–16). They represent the first unambiguous Ordovician SCF assemblages recording diverse animal body plans, comparable to the best-preserved Cambrian counterparts (16, 67, 81, 82) in terms of morphological resolution, completeness, and phylum-level disparity. These SCFs record lightly sclerotized Ordovician taxa in submicron-scale detail, associated with biostratigraphically informative skeletal fossils, and in broadly representative shelf settings across an extensive timeframe, spanning two of the major transgressions that define the early Paleozoic history of Laurentia (*SI Appendix*, Fig. S1).

The capacity of the best Cambrian Konservat-Lagerstätten to reveal (sub)micrometric details even for lightly sclerotized structures (8) contrasts with the coarser preservation of most Ordovician equivalents, where micron-scale characters are typically confined to the most recalcitrant elements (e.g., paleoscolecid plates) (13). By contrast, the submicron-scale resolution and degree of completeness of the Osterberg specimens, which include extensive semiarticulated feeding and cuticular apparatuses (e.g., Figs. 3 n and o, 4 a, y, and z, and 5t), matches that of the most pristine Cambrian SCFs, represented by the Bright Angel fauna of Arizona (16), the Deadwood assemblages of Saskatchewan (19), and the Mount Clark biota of northwestern Canada (83). This high-fidelity record of the Osterberg biotas shows that the post-Cambrian decline of BST macrofossil preservation (7, 9, 12) did not necessarily carry over to microscopic counterparts. Notably, BST preservation at micro- and mesofossil scales—more robust to reworking, bioturbation, and even ingestion (14)—would not have been affected to the same degree as macrofossil equivalents by escalating Ordovician bioturbation and scavenging (84). Therefore, the Osterberg SCFs support increasingly stringent biostratinomic filters as first-order controls on the declining post-Cambrian quality and frequency of (macroscopic) BST fossils, leaving smaller counterparts (14) relatively unaffected.

The high preservation quality of the Osterberg fossils stands against a depositional context unlike that of most early Paleozoic “exceptional” biotas. The epicratonic, subequatorial setting of the Osterberg biotas contrasts with those of major Ordovician Konservat-Lagerstätten (Fig. 6): Notably, these include the higher-latitude, open-shelf to deeper-water Fezouata (11) and Castle Bank (13) biotas and the marginal marine Winneshiek biota—which occupied a restricted embayment lacking typical shelly marine taxa and subject to anoxic sedimentary or bottom conditions (17). By contrast, the paleoenvironment of the Osterberg faunas is directly comparable to those of Cambrian SCF biotas from the well-oxygenated, normal-marine epeiric habitats of Laurentia (14, 63, 67, 83, 85, 86) (Fig. 1). Within this context, the two Osterberg biotas record a chronologically extensive window of exceptional preservation, ranging across two corresponding regional megasequences separated by a major depositional break (Fig. 1 and *SI Appendix*, Fig. S1). This multimillion-year range captures a diverse faunal record in comparatively habitable Ordovician epeiric environments, decoupled from the ecologically stressed or “refugial” settings of most Konservat-Lagerstätten from the same period (Fig. 6).

Against this paleoenvironmental backdrop, the Osterberg biotas capture taxa and adaptations unrecorded by Ordovician macrofossils, but paralleling Cambrian forms from similar shelf settings. The uniqueness of this record is partly attributable to the ability of the Osterberg SCFs to resolve delicate, extremely fine-scale structures. Among these, the submicron-scale, filiform denticles of lower Osterberg priapulid SCFs denote trophic specializations in Ordovician forms but recorded in Cambrian taxa from similar epeiric habitats (16). Finely sculpted “grinding” molars with micrometer-sized scales remain likewise unknown among Ordovician bedding-plane fossils. In the upper Osterberg biota, these specimens capture the next chapter of a radiation of particle-feeding arthropods with Cambrian roots (16, 67, 71), demonstrating a lasting presence of SCF precursors to modern crustacean morphologies in early Paleozoic shelf habitats. Likewise, although shelly fossils record a major Ordovician radiation of molluskan classes (5, 95, 96), their functional morphologies are obscured by a lack of nonmineralized parts—above all their feeding radulae—among coeval macrofossils. By contrast, the lower and upper Osterberg SCFs capture distinct mouthparts comparable to those of early Cambrian specialist herbivores from similar paleoenvironments (45).

The Osterberg fossils support ecological continuity between derived functional guilds first pioneered in Cambrian epeiric seas and increasingly escalated, younger Ordovician assemblages—populated by diverse predatory mollusks, chordates, arthropods, and annelids with sclerotized feeding parts. The producers of “Cambrian-like” grazing radulae from the lower Osterberg biota coexisted with predatory equivalents, unrecorded in other Cambrian to mid-Ordovician deposits. The previous earliest known radulae with multiseriate, hook-like teeth similar to modern carnivorous examples are orthocone specimens from the terminal Ordovician (Hirnantian) (97) Soom Shale. By contrast, the lower Osterberg radulae suggest that predatory mollusks had already diversified in the earliest post-Cambrian ecologies, where they coexisted with typical later-Paleozoic faunal components. In particular, eurypterid-type SCFs in the lower Osterberg biota support a Tremadocian presence of these early Paleozoic chelicerates (11), representing their oldest described occurrence and contributing to reconcile the eurypterid record with the existence of derived members of the group by the Darriwilian (75). Molluskan herbivores and particle-feeding crustaceomorphs from the upper Osterberg biota further demonstrate the co-occurrence of these invertebrates with biomineralized chordate (euconodont) visual predators or planktivores, absent from the prelate Cambrian nekton but ecologically prevalent during the later Paleozoic (1, 33).

The combination of cryptic “Cambrian-style” shelfal SCF faunas and typical Ordovician heavily sclerotized taxa (including graptolites, scolecodonts, eurypterids, and conodonts) denotes a unique faunal complex not captured by Ordovician Konservat-Lagerstätten or shelly assemblages (Fig. 6). By contrast, the Osterberg faunas lack common and readily preservable groups (e.g., wiwaxiids, lobopodians, stem-arthropods) (13, 82, 87, 98) populating coeval deeper-water or refugial habitats (11, 13, 99). This suggests that by the Tremadocian these animals, recorded by iconic sites such as the Fezouata biota (11), were no longer ecologically significant in the resource-rich, physiologically permissive, and geographically widespread shelf settings occupying flooded cratonic interiors during early Paleozoic transgressions (14, 16, 67, 81).

In this light, Ordovician Konservat-Lagerstätten may be unrepresentative of the most habitable and extensive theaters of animal life in their contemporary biosphere. By contrast, the composition of the Osterberg biotas suggests a protracted early Paleozoic segregation between “archaic” faunas in physiologically marginal environments and epeiric “cradles” of modern metazoan groups, first emerging in the Cambrian (14, 16, 82) and sustained well into the Ordovician. Therefore, the most widespread and ecologically escalated Ordovician ecologies may have been markedly more similar to earlier, Cambrian biotas from similar environments than to the “Burgess Shale-like” forms captured by the most iconic, broadly coeval macrofossil faunas (11, 13) (*SI Appendix*, Fig. S7). This early Paleozoic marginalization of Burgess Shale-like faunas provides a proximate, ecological account of the broader stem- and crown-group dynamics reflected by the metazoan fossil record, whereby stem-group taxa tend, by definition, to become extinct relatively soon after the radiation of the crown-group (100, 101).

The Osterberg SCF biotas show that Ordovician epeiric habitats were populated by cryptic, nonskeletonized faunas partly overlapping with those of Cambrian shelf settings, but unrecorded by coeval Konservat–Lagerstätten. Their morphologies bridge the gap between the Cambrian pioneering of modern-style adaptations and their assembly into later-Paleozoic ecologies. Together, the Osterberg biotas showcase the potential of a widespread but underexplored microscopic window of carbonaceous preservation, so far largely confined to the Cambrian, to track the rise of animals well into the later Phanerozoic.

## Materials and Methods

Approximately 50 to 100 g from each sample shown in Fig. 1*C* and Dataset S1 (*SI Appendix*, *Supporting Information Text*) were digested in concentrated hydrofluoric acid. SCFs were hand-picked following the procedure of Butterfield and Harvey (26). Representative specimens were imaged using a Kontron Elektronik ProgRes 3012 camera fitted to a Zeiss Axioplan 2 stereomicroscope or a Quanta-650F scanning electron microscope. Focus stacking was performed in Adobe Photoshop 2024.

## Supplementary Material

Appendix 01 (PDF)

Dataset S01 (XLSX)

## Data Availability

All study data are included in the article and/or supporting information. All figured specimens will be permanently deposited at the National Museum of Natural History, Smithsonian Institution.

## References

[1] T. Servais, A. W. Owen, D. A. T. Harper, B. Kröger, A. Munnecke, The great Ordovician biodiversification event (GOBE): The palaeoecological dimension. Palaeogeogr. Palaeoclimatol. Palaeoecol. **294**, 99–119 (2010).

[2] T. Servais, B. Cascales-Miñana, D. A. T. Harper, The great Ordovician biodiversification event (GOBE) is not a single event. Paleontol. Res. **25**, 315–328 (2021).

[3] A. L. Stigall, C. T. Edwards, R. L. Freeman, C. M. O. Rasmussen, Coordinated biotic and abiotic change during the Great Ordovician Biodiversification Event: Darriwilian assembly of early Paleozoic building blocks. Palaeogeogr. Palaeoclimatol. Palaeoecol. **530**, 249–270 (2019).

[4] J. J. Sepkoski, W. I. Ausich, “Evolutionary faunas” in Palaeobiology: A synthesis, D. E. Briggs, P. R. Crowther, Eds. (Blackwell Scientific Publications, Oxford, 1990), p. 31.

[5] T. Servais , No (Cambrian) explosion and no (Ordovician) event: A single long-term radiation in the early Palaeozoic. Palaeogeogr. Palaeoclimatol. Palaeoecol. **623**, 111592 (2023).

[6] J. Kimmig, J. D. Schiffbauer, A modern definition of fossil-lagerstatten. Trends Ecol. Evol. **39**, 621–624 (2024). 38670863 10.1016/j.tree.2024.04.004

[7] R. R. Gaines, Burgess shale-type preservation and its distribution in space and time. Paleontol. Soc. Pap. **20**, 123–146 (2014).

[8] N. J. Butterfield, Organic preservation of non-mineralizing organisms and the taphonomy of the Burgess Shale. Paleobiology **16**, 272–286 (1990).

[9] F. Saleh , Highly resolved taphonomic variations within the Early Ordovician Fezouata biota. Sci. Rep. **14**, 20807 (2024). 39242693 10.1038/s41598-024-71622-w PMC11379804

[10] F. Saleh , The Cabrières biota (France) provides insights into Ordovician polar ecosystems. Nat. Ecol. Evol. **8**, 651–662 (2024). 38337049 10.1038/s41559-024-02331-w PMC11009115

[11] P. Van Roy, D. E. Briggs, R. R. Gaines, The Fezouata fossils of Morocco; an extraordinary record of marine life in the Early Ordovician. J. Geol. Soc. Lond. **172**, 541–549 (2015).

[12] N. J. Butterfield, Secular distribution of Burgess-shale-type preservation. Lethaia **28**, 1–13 (1995).

[13] J. P. Botting , A middle Ordovician Burgess Shale-type fauna from Castle Bank, Wales (UK). Nat. Ecol. Evol. **7**, 666–674 (2023). 37127766 10.1038/s41559-023-02038-4

[14] N. J. Butterfield, T. H. P. Harvey, Small carbonaceous fossils (SCFs): A new measure of early Paleozoic paleobiology. Geology **40**, 71–74 (2012).

[15] B. Slater, M. S. Bohlin, Animal origins: The record from organic microfossils. Earth-Sci. Rev. **232**, 104107 (2022).

[16] G. Mussini , Evolutionary escalation in an exceptionally preserved Cambrian biota from the Grand Canyon (Arizona, USA). Sci. Adv. **11**, eadv6383 (2025). 40700497 10.1126/sciadv.adv6383 PMC12285723

[17] D. E. G. Briggs, H. B. P. Liu, R. M. Mckay, B. J. Witzke, The Winneshiek biota: Exceptionally well-preserved fossils in a Middle Ordovician impact crater. J. Geol. Soc. Lond. **175**, 865–874 (2018).

[18] H. Nowak, T. H. P. Harvey, H. B. P. Liu, R. M. McKay, T. Servais, Exceptionally preserved arthropodan microfossils from the Middle Ordovician Winneshiek Lagerstatte, Iowa, USA. Lethaia **51**, 267–276 (2018).

[19] T. H. Harvey, M. I. Vélez, N. J. Butterfield, Small Carbonaceous Fossils from the Earlie and Deadwood Formations (Middle Cambrian to Lower Ordovician) of Southern Saskatchewan (Saskatchewan Geological Survey, 2012), pp. 1–8.

[20] C. Lochman-Balk, J. L. Wilson, Stratigraphy of upper Cambrian-lower Ordovician subsurface sequence in Williston Basin. AAPG Bull. **51**, 883–917 (1967).

[21] C. Lochman, Upper Cambrian faunas from the subsurface Deadwood Formation, Williston Basin. J. Paleontol. **38**, 33–60 (1964).

[22] J. B. Ellingson, “Depositional environments and paleogeography of the Winnipeg Group (Ordovician), Williston Basin, North Dakota,” Master’s Thesis, University of North Dakota, Grand Forks, North Dakota (1995), p. 78.

[23] A. H. Sarnoski Jr., “The stratigraphy and depositional history of the Deadwood Formation, with a focus on early Paleozoic subsidence in the Williston Basin,” Master’s Thesis, University of North Dakota, Grand Forks (2015).

[24] D. B. Anderson, “Stratigraphy and depositional history of the deadwood formation (upper Cambrian and lower Ordovician), Williston Basin, North Dakota,” Master’s Thesis, University of North Dakota, Grand Forks (1988), p. 330.

[25] F. J. Hein, G. S. Nowlan, Regional sedimentology, conodont biostratigraphy and correlation of Middle Cambrian-Lower Ordovician (?) strata of the “Finnegan” and Deadwood formations, Alberta subsurface, Western Canada Sedimentary Basin. Bull. Can. Pet. Geol. **46**, 166–188 (1998).

[26] S. C. Thompson, “Depositional environments and history of the Winnipeg Group (Ordovician), Williston Basin, North Dakota,” Master’s Thesis, University of North Dakota, Grand Forks, North Dakota (1984), p. 295.

[27] J. W. Bader, “Stratigraphic framework of the deadwood formation, North Dakota” in Geologica Investigation No. 266, E. C. Murphy, L. D. Helms, Eds. (North Dakota Geological Survey, Bismarck, North Dakota, 2022).

[28] C. Lochman, Lower ordovician (arenig) faunas from the Williston Basin, Montana and North Dakota. J. Paleontol. **40**, 512–548 (1966).

[29] C. Lochman, Basal Ordovician faunas from the Williston Basin, Montana. J. Paleontol. **38**, 453–476 (1964).

[30] J. W. Bader, J. H. Lake, A. H. Sarnoski, “Deadwood formation (Cambrian/Ordovician) of North Dakota: A core Atlas” in Geologic Investigation No. 265, E. C. Murphy, L. D. Helms, Eds. (North Dakota Geological Survey, Bismarck, North Dakota, 2022).

[31] W. C. Sweet, Conodonts from the Winnipeg Formation (Middle Ordovician) of the northern Black Hills, South Dakota. J. Paleontol. **56**, 1029–1049 (1982).

[32] J. Dorador, L. A. Buatois, M. G. Mángano, F. J. Rodríguez-tovar, Ichnology of the Winnipeg Formation, southeast Saskatchewan: A glimpse into the marine infaunal ecology of the Great Ordovician Biodiversification Event. Lethaia **52**, 14–30 (2019).

[33] W. C. Sweet, P. C. J. Donoghue, Conodonts: Past, present, future. J. Paleontol. **75**, 1174–1184 (2001).

[34] J. A. Bauer, Conodonts and conodont biostratigraphy of the joins and oil creek formations, Arbuckle Mountains, South-central Oklahoma. Okla. Geol. Surv. Bull. **150**, 0078–4389 (2010).

[35] J. A. Bauer, Conodont biostratigraphy and paleoecology of middle Ordovician rocks in eastern Oklahoma. J. Paleontol. **63**, 92–107 (1989).

[36] J. A. Bauer, Conodonts and conodont biostratigraphy of the Mclish and Tulip Creek formations (Middle Ordovician) of south-central Oklahoma. Okla. Geol. Surv. Bull. **141**, 0078–4397 (1987).

[37] R. L. Ethington, D. L. Clark, Lower and middle Ordovician conodonts from the Ibex area, Western Millard County, Utah. Brigham Young Univ. Geol. Stud. **28**, 1–160 (1982).

[38] H. P. Liu , Exceptionally preserved conodont apparatuses with giant elements from the Middle Ordovician Winneshiek Konservat-Lagerstatte, Iowa, USA. J. Paleontol. **91**, 493–511 (2017).

[39] M. C. Mound, Two new conodont genera from the Joins Formation (lower Middle Ordovician) of Oklahoma. Biol. Soc. Wash. Proc. **78**, 193–200 (1965).

[40] E. Beli, S. Piraino, C. B. Cameron, Fossilization processes of graptolites: Insights from the experimental decay of Rhabdopleura sp. (Pterobranchia). Palaeontology **60**, 389–400 (2017).

[41] R. Lerosey-Aubril , Benthic pterobranchs from the Cambrian (Drumian) Marjum Konservat-Lagerstätte of Utah. Pap. Palaeontol. **10**, e1555 (2024).

[42] J. Maletz, “The planktic revolution” in Graptolite Paleobiology, B. L. Krebs, Ed. (Wiley Blackwell, Hoboken, NJ, 2017), pp. 139–152.

[43] S. C. Finney, W. B. Berry, New perspectives on graptolite distributions and their use as indicators of platform margin dynamics. Geology **25**, 919–922 (1997).

[44] J. Maletz, X. F. Wang, C. S. Wang, Early tremadocian (Ordovician) graptolite biostratigraphy and correlation. Palaeoworld **32**, 44–62 (2023).

[45] B. Slater, Life in the Cambrian shallows: Exceptionally preserved arthropod and mollusk microfossils from the early Cambrian of Sweden. Geology **52**, 256–260 (2024).

[46] B. J. Slater, Cambrian “sap-sucking” molluscan radulae among small carbonaceous fossils (SCFs). Proc. R. Soc. B Biol. Sci. **290**, 20230257 (2023). 10.1098/rspb.2023.0257 PMC10050940 36987646

[47] P. J. Krug, J. E. Vendetti, A. Valdés, Molecular and morphological systematics of Elysia Risso, 1818 (Heterobranchia: Sacoglossa) from the Caribbean region. Zootaxa **4148**, 1–137 (2016). 27515641 10.11646/zootaxa.4148.1.1

[48] D. Montroni , Structural characterization of the buccal mass of *Ariolimax californicus* (Gastropoda; Stylommatophora). PLoS One **14**, e0212249 (2019). 31390363 10.1371/journal.pone.0212249 PMC6685607

[49] G. Pastorino, F. Scarabino, Two new deep-sea muricids (Gastropoda) from Argentina. Nautilus **122**, 107–114 (2008).

[50] N. Gajera, B. Vakani, R. Kundu, Radular morphology and relationship between shell size and radula size of few dominating intertidal gastropod Mollusks of Veraval Coast, Gujarat. Front. Mar. Sci. **9**, 657124 (2022).

[51] V. O. Barkalova, A. E. Fedosov, Y. I. Kantor, Morphology of the anterior digestive system of tonnoideans (Gastropoda: Caenogastropoda) with an emphasis on the foregut glands. Molluscan Res. **36**, 54–73 (2016).

[52] S. E. Gabbott, Orthoconic cephalopods and associated fauna from the late Ordovician Soom Shale Lagerstatte, South Africa (vol 42, pg 140, 1999). Palaeontology **42**, 567–568 (1999).

[53] I. Kruta, N. H. Landman, K. Tanabe, “Ammonoid radula” in Ammonoid Paleobiology: From Anatomy to Ecology, C. Klug, D. Korn, K. De Baets, I. Kruta, R. H. Mapes, Eds. (Topics in Geobiology, Springer, New York, 2015), **vol. 43**.

[54] V. Russini , Whelks, rock-snails, and allied: A new phylogenetic framework for the family Muricidae (Mollusca: Gastropoda). Eur. Zool. J. **90**, 856–868 (2023).

[55] E. E. Strong, N. Puillandre, A. G. Beu, M. Castelin, P. Bouchet, Frogs and tuns and tritons—A molecular phylogeny and revised family classification of the predatory gastropod superfamily Tonnoidea (Caenogastropoda). Mol. Phylogenet. Evol. **130**, 18–34 (2019). 30278253 10.1016/j.ympev.2018.09.016

[56] N. J. Butterfield, An early Cambrian radula. J. Paleontol. **82**, 543–554 (2008).

[57] G. Rouse, F. Pleijel, Polychaetes (Oxford University Press, Oxford (UK), 2001).

[58] B. J. Slater, T. H. P. Harvey, R. Guilbaud, N. J. Butterfield, A cryptic record of Burgess shale-type diversity from the Early Cambrian of Baltica. Palaeontology **60**, 117–140 (2017).

[59] B. Slater, S. Willman, Early cambrian small carbonaceous fossils (SCFs) from an impact crater in western Finland. Lethaia **52**, 570–582 (2019).

[60] G. Giribet, G. D. Edgecombe, Current understanding of Ecdysozoa and its internal phylogenetic relationships. Integr. Comp. Biol. **57**, 455–466 (2017). 28957525 10.1093/icb/icx072

[61] A. Schmidt-Rhaesa, “Priapulida” in Handbook of Zoology: Gastrotricha, Cycloneuralia and Gnathifera. Vol. 1: Nematomorpha, Priapulida, Kinorhynca, Loricifera, A. Schmidt-Rhaesa, Ed. (De Gruyter, Berlin, 2012).

[62] M. R. Smith, T. H. P. Harvey, N. J. Butterfield, The macro- and microfossil record of the Cambrian priapulid *Ottoia*. Palaeontology **58**, 705–721 (2015).

[63] G. Mussini, Y. P. Veenma, N. J. Butterfield, A peritidal burgess-shale-type fauna from the middle Cambrian of western Canada. Palaeontology **68**, e70001 (2025).

[64] J. V. Wernström, B. J. Slater, M. V. Sorensen, D. Crampton, A. Altenburger, Geometric morphometrics of macro- and meiofaunal priapulid pharyngeal teeth provides a proxy for studying Cambrian “tooth taxa”. Zoomorphology **142**, 411–421 (2023).

[65] M. V. Sorensen, H. S. Rho, W. G. Min, D. Kim, A new recording of the rare priapulid Meiopriapulus fijiensis, with comparative notes on juvenile and adult morphology. Zool. Anz. **251**, 364–371 (2012).

[66] G. D. Edgecombe, S. Richter, G. D. Wilson, The mandibular gnathal edges: Homologous structures throughout Mandibulata? Afr. Invertebr. **44**, 115–135 (2003).

[67] T. H. P. Harvey, M. I. Vélez, N. J. Butterfield, Exceptionally preserved crustaceans from western Canada reveal a cryptic Cambrian radiation. Proc. Natl. Acad. Sci. U.S.A. **109**, 1589–1594 (2012). 22307616 10.1073/pnas.1115244109 PMC3277126

[68] P. Taugourdeau, Débris microscopiques d’euryptéridés du Paléozoïque Saharien. Rev. Micropaleontol. **10**, 119–127 (1967).

[69] A. Makled , Eurypterid setae and cuticle fragments from the Ora Formation (Upper Devonian) of Iraq. Palynology **49**, 2445034 (2025).

[70] G. Mura, Morphological features of the mandible related to feeding habits of some Anostraca species. Crustaceana **68**, 83–102 (1995).

[71] T. H. P. Harvey, N. J. Butterfield, A new species of early Cambrian arthropod reconstructed from exceptionally preserved mandibles and associated small carbonaceous fossils (SCFs). Pap. Palaeontol. **8**, e1458 (2022).

[72] T. A. Hegna, J. Luque, J. M. Wolfe, “The fossil record of the Pancrustacea” in Evolution and Biogeography, M. Thiel, G. Poore, Eds. (The Natural History of the Crustacea, Oxford University Press, Oxford, 2020), **vol. 8**.

[73] A. I. Camacho, A. G. Valdecasas, Global diversity of syncarids (Syncarida; Crustacea) in freshwater. Hydrobiologia **595**, 257–266 (2008).

[74] G. Mura, Scanning electron microscopic study of the molar surfaces of the mandibles of *Chirocephalus diaphanus* Prévost (Anostraca). Crustaceana **60**, 178–185 (1991).

[75] J. C. Lamsdell, D. E. G. Briggs, H. B. P. Liu, B. J. Witzke, R. M. Mckay, The oldest described eurypterid: A giant Middle Ordovician (Darriwilian) megalograptid from the Winneshiek Lagerstatte of Iowa. BMC Evol. Biol. **15**, 169 (2015). 26324341 10.1186/s12862-015-0443-9 PMC4556007

[76] P. Filipiak, M. Zaton, Plant and animal cuticle remains from the Lower Devonian of southern Poland and their palaeoenvironmental significance. Lethaia **44**, 397–409 (2011).

[77] P. Filipiak, P. Kenrick, Z. Wawrzyniak, M. Kondas, C. Strullu-Derrien, Plants and palynomorphs from the Lower Devonian (upper Emsian) of the Holy Cross Mountains, Poland. Rev. Palaeobot. Palynol. **302**, 104666 (2022).

[78] P. Filipiak, M. Zaton, H. Szaniawski, R. Wrona, G. Racki, Palynology and microfacies of lower Devonian mixed carbonate-siliciclastic deposits in Podolia, Ukraine. Acta Palaeontol. Pol. **57**, 863–877 (2012).

[79] P. L. Manning, J. A. Dunlop, The respiratory organs of eurypterids. Palaeontology **38**, 287–297 (1995).

[80] J. B. Hou, N. C. Hughes, M. J. Hopkins, The trilobite upper limb branch is a well-developed gill. Sci. Adv. **7**, eabe7377 (2021). 33789898 10.1126/sciadv.abe7377 PMC8011964

[81] T. H. P. Harvey, N. J. Butterfield, Sophisticated particle-feeding in a large Early Cambrian crustacean. Nature **452**, 868–871 (2008). 18337723 10.1038/nature06724

[82] G. Mussini, N. J. Butterfield, A microscopic Burgess Shale: Small carbonaceous fossils from a deeper water biota and the distribution of Cambrian non-mineralized faunas. Proc. R. Soc. B Biol. Sci. **292**, 20242948 (2025). 10.1098/rspb.2024.2948 PMC11836709 39968618

[83] T. H. P. Harvey, N. J. Butterfield, Great Canadian lagerstatten 2. Macro- and microfossils of the mount cap formation (early and middle Cambrian, Northwest Territories). Geosci. Can. **38**, 165–173 (2011).

[84] R. R. Gaines , Burgess Shale-type biotas were not entirely burrowed away. Geology **40**, 283–286 (2012).

[85] B. J. Slater, S. Willman, G. E. Budd, J. S. Peel, Widespread preservation of small carbonaceous fossils (SCFs) in the early Cambrian of North Greenland. Geology **46**, 107–110 (2018).

[86] E. Wallet, B. Slater, S. Willman, J. S. Peel, Small carbonaceous fossils (SCFs) from North Greenland: New light on metazoan diversity in early Cambrian shelf environments. Pap. Palaeontol. **7**, 1403–1433 (2021).

[87] J. P. Botting, L. A. Muir, N. Jordan, C. Upton, An ordovician variation on Burgess Shale-type biotas. Sci. Rep. **5**, 9947 (2015). 25909638 10.1038/srep09947 PMC4408981

[88] F. Saleh , The chengjiang biota inhabited a deltaic environment. Nat. Commun. **13**, 1569 (2022). 35322027 10.1038/s41467-022-29246-z PMC8943010

[89] K. Nanglu, J. B. Caron, R. R. Gaines, The burgess shale paleocommunity with new insights from Marble Canyon, British Columbia. Paleobiology **46**, 58–81 (2020).

[90] X. Fang , The Liexi fauna: A new Lagerstatte from the Lower Ordovician of South China. Proc. R. Soc. B Biol. Sci. **289**, 20221027 (2022). 10.1098/rspb.2022.1027 PMC9277276 35858062

[91] T. H. P. Harvey, J. Ortega-Hernández, J. P. Lin, Y. L. Zhao, N. J. Butterfield, Burgess shale-type microfossils from the middle Cambrian Kaili Formation, Guizhou Province, China. Acta Palaeontol. Pol. **57**, 423–436 (2012).

[92] Y. L. Zhao , Kaili biota: A taphonomic window on diversification of metazoans from the basal middle Cambrian: Guizhou, China. Acta Geol. Sin. Engl. **79**, 751–765 (2005).

[93] D. A. T. Harper , The Sirius Passet Lagerstatte of North Greenland: A remote window on the Cambrian explosion. J. Geol. Soc. Lond. **176**, 1023–1037 (2019).

[94] J. R. Foster, R. R. Gaines, Taphonomy and paleoecology of the ‘middle’ Cambrian (Series 3) formations in Utah’s West Desert: Recent finds and new data. Utah Geol. Assoc. Publ. **45**, 291–336 (2016).

[95] P. M. Novack-Gottshall, A. I. Miller, Comparative geographic and environmental diversity dynamics of gastropods and bivalves during the Ordovician radiation. Paleobiology **29**, 576–604 (2003).

[96] B. Kröger, T. Servais, Y. B. Zhang, The origin and initial rise of pelagic cephalopods in the Ordovician. PLoS One **4**, e7262 (2009). 19789709 10.1371/journal.pone.0007262 PMC2749442

[97] S. E. Gabbott, C. Browning, J. N. Theron, R. J. Whittle, The late Ordovician Soom Shale Lagerstatte: An extraordinary post-glacial fossil and sedimentary record. J. Geol. Soc. London **174**, 1–9 (2017).

[98] J. B. Caron, M. R. Smith, T. H. P. Harvey, Beyond the Burgess Shale: Cambrian microfossils track the rise and fall of hallucigeniid lobopodians. Proc. R. Soc. B Biol. Sci. **280**, 20131613 (2013). 10.1098/rspb.2013.1613 PMC3735267 23902914

[99] J. Kimmig, H. Couto, W. W. Leibach, B. S. Lieberman, Soft-bodied fossils from the upper Valongo Formation (Middle Ordovician: Dapingian-Darriwilian) of northern Portugal. Sci. Nat. **106**, 27 (2019). 10.1007/s00114-019-1623-z 31129730

[100] G. E. Budd, R. P. Mann, The dynamics of stem and crown groups. Sci. Adv. **6**, eaaz1626 (2020). 32128421 10.1126/sciadv.aaz1626 PMC7030935

[101] G. E. Budd, R. P. Mann, Survival and selection biases in early animal evolution and a source of systematic overestimation in molecular clocks. Interface Focus **10**, 20190110 (2020). 32637066 10.1098/rsfs.2019.0110 PMC7333906

