# Supplementary material for: Two exceptionally preserved biotas from North Dakota reveal cryptic Ordovician shelf ecologies
Source: Proc Natl Acad Sci U S A. 2025 Nov 3;122(46):e2520246122. doi: 10.1073/pnas.2520246122 (PMC12646318; doi:10.1073/pnas.2520246122)
Supplement: Supplementary file 1 — Appendix 01 (PDF) [file pnas.2520246122.sapp.pdf]

PNAS

Supporting Information for

Two exceptionally preserved biotas from North Dakota (USA) reveal cryptic Ordovician shelf ecologies

Giovanni Mussini and Nicholas J. Butterfield

Corresponding author: Giovanni Mussini
Email: gm726@cam.ac.uk

This PDF file includes:

Supporting text
Figures S1 to S7
Legend for Dataset S1
SI References

Other supporting materials for this manuscript include the following:

Dataset S1

Supporting Information Text

Metazoan SCFs from the Osterberg biotas: Supplementary descriptions

Other conodonts

Beyond the abundant *Multioistudus* and *?Neomultioistodus*, sparser conodonts from the upper Osterberg biota (N=107; Data S1), allowing for more preliminary identification and potentially including new taxa, suggest a coherent plexus of mid-Ordovician Laurentian genera. *Erraticodon* (1) is recorded by dolabrate M elements with long posteriorly curved cusps and laterally compressed denticles on a posterior keel [(Fig. S3o-q, ab cf. (2), text fig. 6B, D, pl. 2 fig. 17; (1), fig. 4C; (3) fig. 6H)], and potentially by alate Sa elements with elongate, gently recurved cusps [(Fig. S3ncf. (1), fig. 7E; (2), pl. 2 fig. 14)]; pastinate Pa elements with anterior and posterior denticulate processes [(Fig. S3s cf. (2) text fig. 6A, pl. 2 fig. 18)]; and Sd-like elements [(Fig. S3rcf. (4) fig. 7F-M)]. *Baltoniodus* is represented by pastinate teeth with extensive, asymmetrical lateral denticulate processes, matching Pb elements in the genus [(Fig. S3d-e, ae cf. (5), fig. 3D; (6) fig. 3.3; (7) fig. 6B)].

Additional, minor components of the Osterberg assemblage include *Acontiodus*-like coniform teeth [Fig. S3f, t cf. (8)]; pastinate, laterally compressed teeth with a weakly arched base, a posteriorly tilted cusp, and compressed lateral and posterior processes with basally crowded denticles (Fig. S3c, ac-ad), comparable to P elements in the cosmopolitan mid- to late-Ordovician *Phragmodus* (2, 9, 10); and multicuspidate to serrated bladelike teeth (Fig. S3a-b, af-ag) similar to P [(11), figs. 17N, 16B-C, 20L] and S [(11), fig. 16D] elements in *Plectodina* [(12), fig. 7]. These specimens co-occur with undescribed cuspidate, subtriangular elements reminiscent of single-element “*Trichonodella”* [(13) pl. VI fig. 18] and “*Prioniodus navis*” [(13) pl. V fig. 35] teeth, but differing in their presence of adenticulate lateral processes (Fig. S3h-m, u-y).

Other arthropods

A 170 µm-long cuticular fragment recovered from the Deadwood Formation (lower Osterberg biota), bearing robust spines of varying length (5-20 µm) on one margin (Fig. 5e), is comparable to the gnathobases of eurypterid coxae [cf. (14), fig. 8D; (15), fig. 6]. However, the morphologically disparate spinose gnathobases of Palaeozoic to Recent chelicerates [e.g. (15, 16)] caution against conclusive attributions. Other ‘serrated’ SCFs of potential arthropod provenance, recovered in the lower Osterberg biota, include a ~400 µm-long process bearing robust, subtriangular uniseriate teeth, comparable to the rami of eurypterid chelicerae [Fig. S5h, cf. (17), fig. 10] or the spinose edge of gnathobases in other chelicerates, some of which bear teeth with a similar fibrous construction (cf. Fig. S5h’) suited for durophagy [cf. (16, 18)].

A more delicate but extensively articulated SCF from Deadwood strata (lower Osterberg biota) consists of a series of subtriangular 'feathery' plates. The entire series is approximately 200 µm long and 40 µm tall (Fig. 5t). Each feathery plate has a laterally asymmetrical profile, arching markedly to one side and tapering to a spindle-like basal 'saddle' on its convex margin. The concave margin bears a series of setulose filaments, longer distally (Fig. 5t'-t''). The feathery plates are united basally by a strip of semi-transparent cuticle. Series of feathery cuticular structures or lamellae constitute molluscan gills (ctenidia). However, the lamellae of ctenidia are basally fused and lack the pronounced asymmetries of the Osterberg specimen [(19), fig. 9; (20), fig. 1]. Closer counterparts are respiratory organs from trilobite upper limb branches, whose closely packed, basally distinct projections arch laterally and taper distally as in the Osterberg SCF [(21), fig. 1].

Non-diagnostic scalidophoran elements

The diagnostic lower Osterberg pharyngeal teeth co-occur with falcate sclerites bearing biseriate denticles on their concave margin, identified as priapulid introvert hooks (Fig. 4k, x, aa) (22, 23). Also attributable to priapulids are characteristically hollow spines (24, 25) bearing the same 'scaly' patterned cuticles (Fig. 4b) found attached to *Ottoia*-type teeth (22, 26). Slender, 50-200 µm-long edentulous hooks with a well-delineated base, sitting on shoe-shaped pads of delicate polygonally patterned cuticle (Fig. 4c-j), closely resemble trunk sclerites from putative stem-group kinorhynchs [(27), fig. 3h-i], also suggesting a scalidophoran producer.

Problematica and non-metazoan SCFs from the Osterberg biotas

Together with 2181 elements showing diagnostic traits of recognisable animal phyla, the Osterberg SCFs includes 1935 taxonomically problematic specimens and elements of probable non-metazoan origin (Data S1; Fig. S2).

20-90 µm-wide translucid, thin-walled and subspherical SCFs, occurring throughout the lower and upper Osterberg biotas, are identifiable as leiosphaerid acritarchs (N = 441), which occur widely among previously documented SCF biotas (18, 26, 28-33). These elements are smooth-walled, but some specimens show concentric creasing patterns (Fig. S2a-g).

Rarer non-metazoan SCFs, recovered in the lower Osterberg biota, include tangled mats (N = 3) of thin, filamentous organic strands attributable to the form taxon *Syphonophycus*, recording probable cyanobacterial producers (33) (Fig. S2h): morphologically similar specimens have been widely documented in shallow-marine Cambrian SCF biotas, and are consistent with a depositional environment within the photic zone (18, 26, 33). Additional filamentous forms from the lower Osterberg biota, up to 700 µm long, consist of smooth, pseudo-segmented strap-shaped envelopes encasing unbranched trichomes (Fig. S2p).

These filaments are morphologically referrable to the form-taxon *Palaeolyngbya* (34). Filament-like, photosynthetic producers are also consistent with a range of wide semi-transparent, strap-shaped, and occasionally false-branching sheets (Fig. S2k-l) occurring throughout the Osterberg drillcores (Data S1). The shapes, large size (up to 500 µm long), and optical density of these fossils are similar to those of broad sinuous filaments assigned to the form taxon *Palaeosiphonella* [e.g. (33), pl. 6]. By contrast, co-occurring semi-tubular carbonaceous fragments occasionally showing false-branching patterns and circular cross-sections (Fig. S2q-s) may record multicellular filamentous algae [cf. (35), fig. 2; (36) fig. 1H].

Other morphologically distinctive SCFs, recovered in the lower Osterberg biota, are carbonaceous sheets (N = 13) approximately 100-200 µm wide and consisting of a system of strictly parallel fibres with adhering (but occasionally 'unstuck'; Fig. S1m, o) lateral edges. Clean breaks within the fibres and conspicuously frayed edges denote a relatively sinewy, recalcitrant matrix. These fossils are optically denser than co-occurring acritarchs and other thin-walled fossils, and adorned by densely spaced, minute dark punctae (e.g. Fig. S1o'). These SCFs occasionally show feather-like protrusions running along one of their edges (Fig. S1n). The protrusions are adorned by densely bundled, pinnule-shaped elements, longest distally (Fig. S1n').

Another category of abundant (N = 99) problematic SCFs from the lower Osterberg biota consists of c. 150-200 µm-long 'club-like' elements (Fig. S2t-w). These thin-walled organic fossils show drum-shaped terminations at the opposite ends of a constricted, U-shaped tubular body. The 'drums', c. 30-60 µm-wide, are unequal in size, with one up to one-third wider than the other; both express a slight central depression and patterns of widely spaced radial creases (Fig. S2u-w). These forms differ from other documented Recent and early Palaeozoic U-shaped algae in their broadened, non-tapering terminations, deviating from a simple crescentic outline [see (18); cf. (18), fig. 4u].

In addition to these morphologically distinctive SCFs, the lower and upper Osterberg specimens comprise carbonaceous fragments varying in morphology from thin, semi-translucent unornamented sheets (Fig. S2j) to thicker cuticles marked by conspicuous longitudinal creases (Fig. S2x) and smooth fragments continuous with unornamented tubes (Fig. S2y). Pending the discovery of more complete material, these forms are considered *incertae sedis*.

Supplementary Materials and Methods

The fossils described in the present study were extracted from greenish-grey shales sampled from the Osterberg 21-2 (48.802903, -101.642301) and Osterberg 22X (48.802904, -101.642302) oil exploration wells in central-northern North Dakota (Renville County), from depths of 9124 to 9297 feet (c. 2780-2833 m; Fig. 1a-b; Data S1).

A total of 38 shale samples were collected for microfossil analysis, 14 from Osterberg 21-2 and 24 from Osterberg 22X (Fig. 1c). Of these, 31 samples were from the Deadwood Formation (12 from 21-2 and 19 from 22X), spanning approximately 54 metres of members B-C. Lithologically, the sampled section of the Deadwood is dominated by bioturbated siltstone (member B) and sandstone (member C) with interspersed shales and conglomerates (Fig. 1c). Of the Deadwood samples, all except one yielded SCFs, and four of these yielded pelagic graptolites (Fig. 1c; see *Graptolites*). 7 samples (4 from the 22X well, 3 from the 21-2 well) from white sandstone-dominated horizons, spanning c. 4 metres of the overlying Winnipeg Formation, were also processed, yielding SCFs and conodonts (Fig. 1c). Therefore, the Osterberg drillcores span sedimentary packages deposited during both the Sauk (Deadwood members B-C) and Tippecanoe (Winnipeg Formation) megasequences. The two drillcores show matching wireline logs and lithology and host directly correlatable conodont and graptolite faunas (Fig. 1c), but yielded no macroscopic shelly fossils (119).

The presence of *Multioistodus* and a consistent assemblage of coeval conodonts identifies the sampled strata of the Winnipeg Formation as Darriwilian (468,1 ± 1,6–460,9 ± 1,6 Ma) and younger than the Sandbian (458.2 ± 0.7–452.8 ± 0.7 Ma) Icebox and Roughlock members (48). Accordingly, the lithology of white sandstone with occasionally dense shaly interbeds characterising the Winnipeg horizons of the Osterberg drillcores is typical of the Black Island member (48, 119, 120).

Supporting Figures

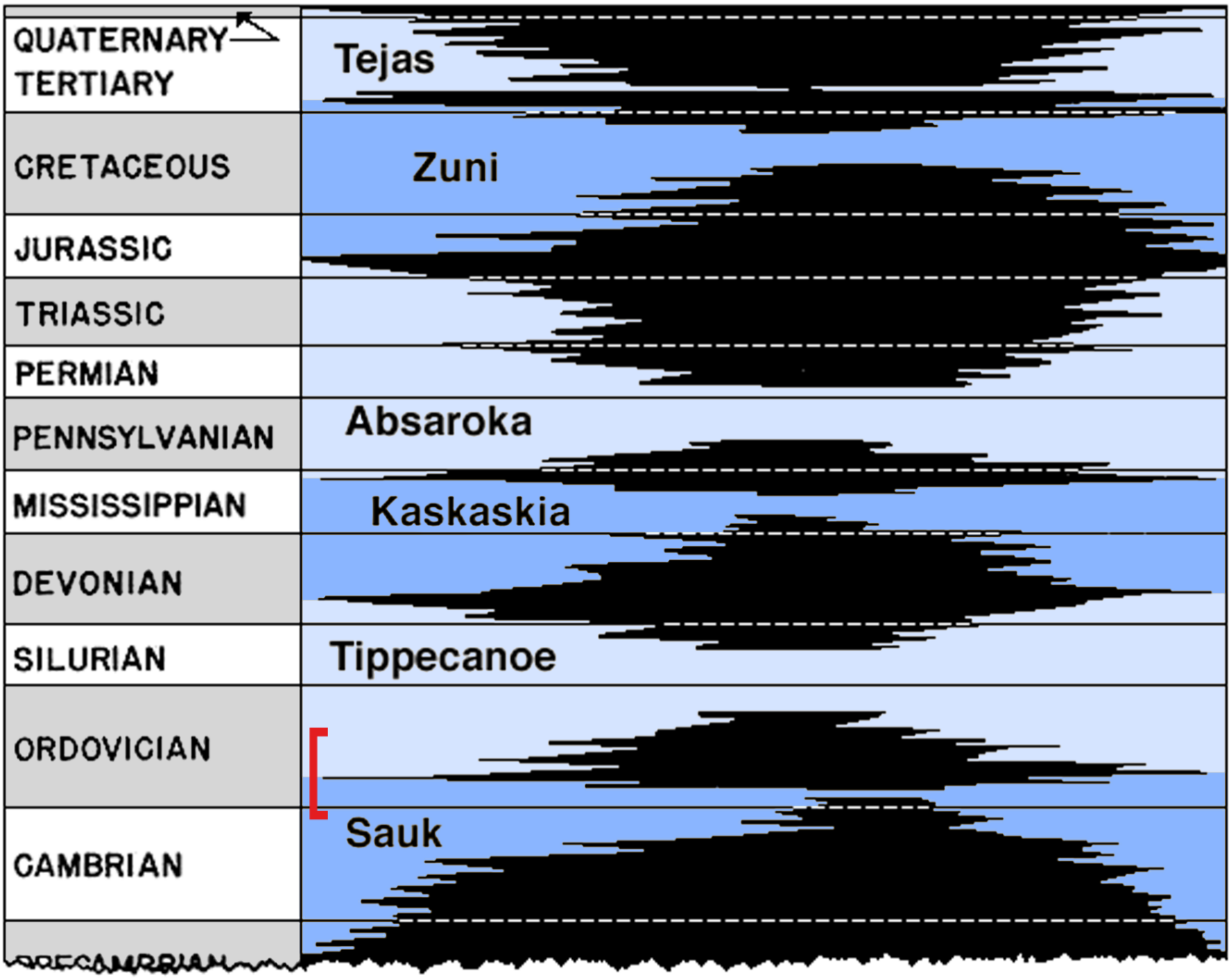

Fig. S1: The record of major marine transgressions on the western North American craton. Time-stratigraphic relationships of sequences in the North American craton from the Precambrian to the Recent, after (37). Black areas represent non-depositional hiatuses; blue areas denote successive alternating sequences of deposition. Transgression names are indicated on the diagram. Approximate temporal extremes of the deposition of the two Osterberg biotas are denoted by the red bracket on the left.

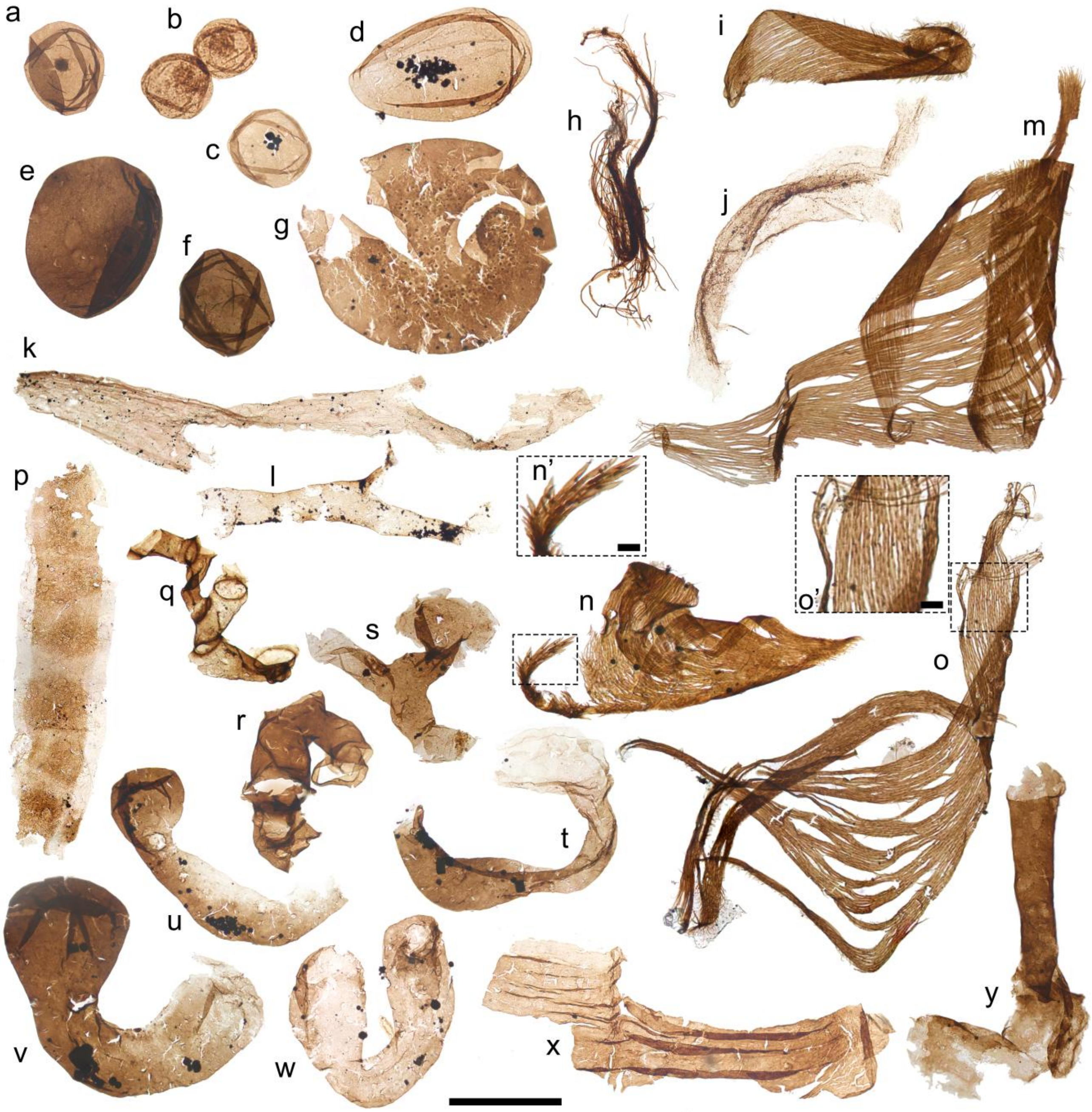

Fig. S2. Non-metazoans and problematic microfossils from the lower (a-d, f-w, y) and upper (e, x) Osterberg biotas. a-g, sphaeroidal acritarchs. **h**, *Syphonophycus*-type filamentous aggregate. **i, m-o**, fibrous sheets showing details of 'feathery' terminations (n') and optically dense punctae (o'). **j**, smooth semi-transparent cuticular sheet. **k-l**, false-branching filaments, cf. *Palaeosiphonella*. **p**, *Palaeolyngbya*-type pseudo-segmented strap. **q-s**, possible algal fragments showing false branching and circular cross-sections. **t-w**, probable algae with drum-shaped opposite termination. **x**, sheet-like fragment with longitudinal creases. **y**, thin-walled tubular problematicum. Scale bars: 50 μm except for k-l, x (100 μm), p (200 μm). Slide numbers and England Finder coordinates given in Data S1.

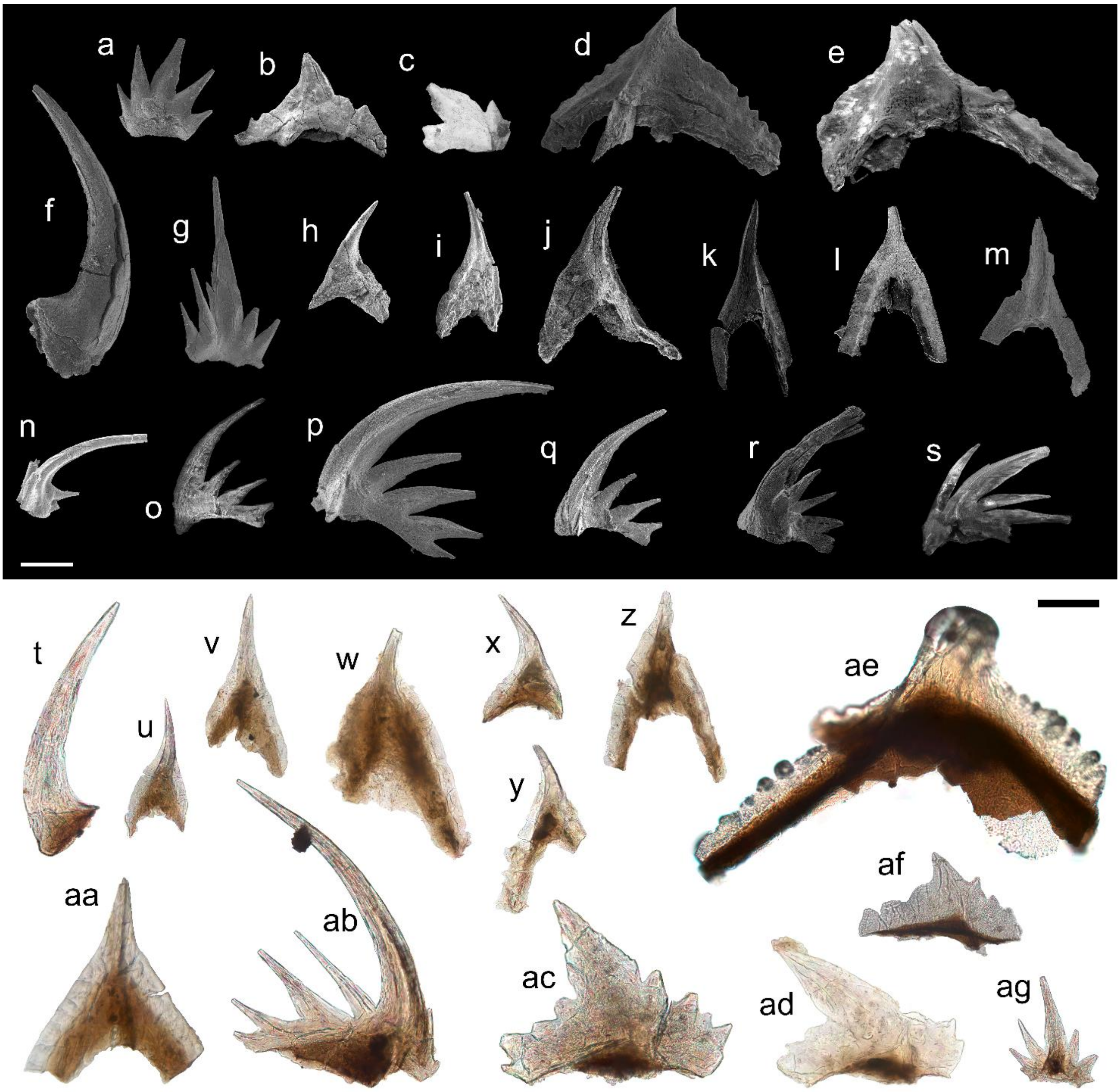

Fig. S3 Additional conodonts from the upper Osterberg biota. a-b, *?Plectodina sp*. a, ?P element; b, ?S element. **c**, cf. *Phragmodus* sp. P element. **d-e**, *Baltoniodus* sp. Pb elements. **f**, coniform element, cf. *Acontiodus*. **g**, *?Plectodina sp*. (cf. a). **h-m**, transition series of adenticulate cuspidate elements. **n-s**, *Erraticodon* sp. n, Sa element; o-q, cf. M elements; r, ?Sd element; s, ?Pa element. a-s photographed using SEM imaging. **u-aa**, transition series of adenticulate cuspidate elements. **ac-ad**, *Phragmodus* sp., P elements. **ae**, *Baltoniodus* sp., Pb element. **t**, coniform element, cf. *Acontiodus*. **af-ag**, *?Plectodina sp*.; ag, ?S element; af, ?P element. Scale bars: 100 µm except for n (200 µm). Slide numbers and England Finder coordinates given in Data S1.

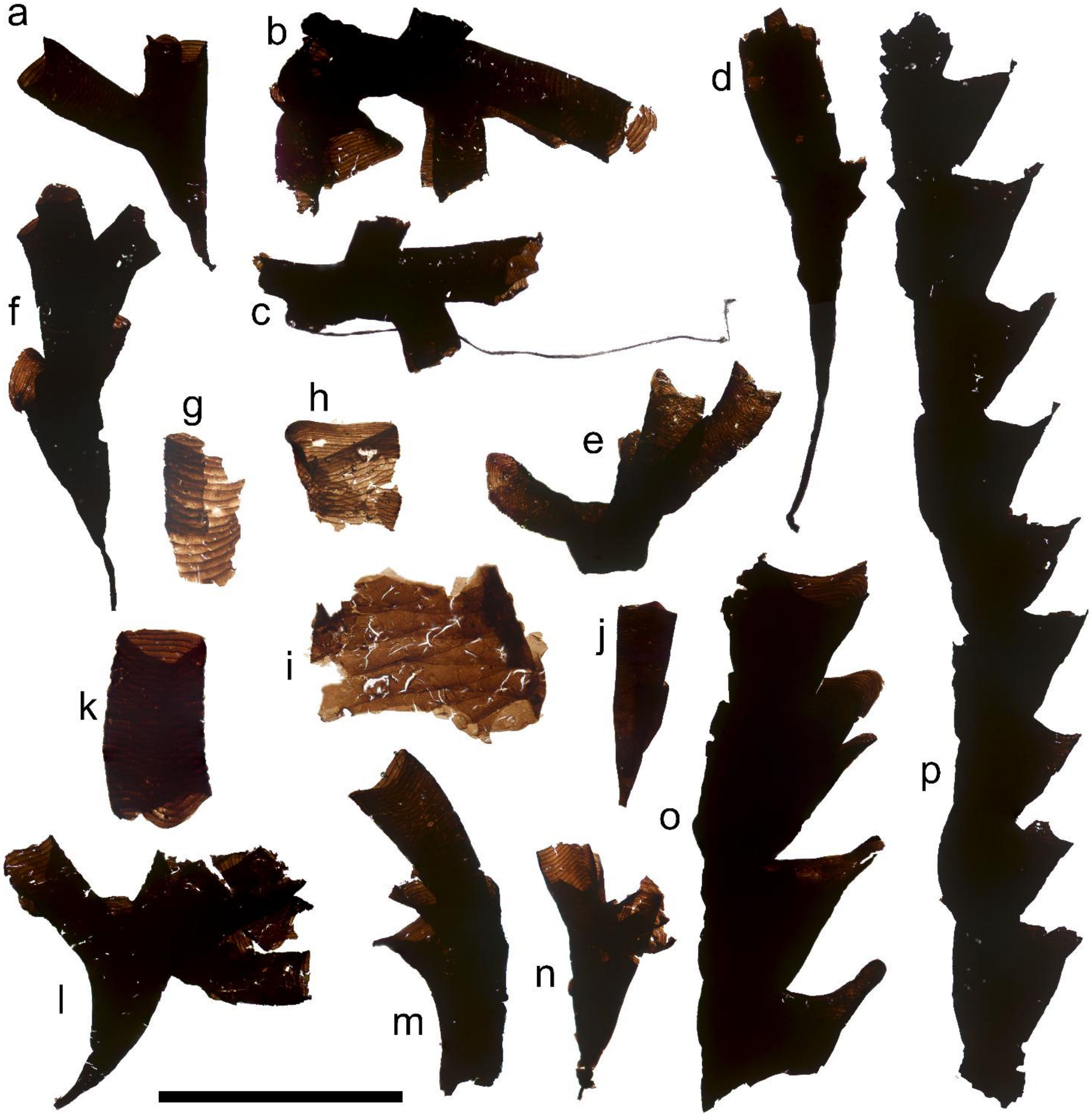

Fig. S4. Additional pterobranchs from the lower Osterberg biota. **a**, **l**, **n**, branching basal portions of graptolite tubaria with tapering siculae. **b**, **e**, branching specimens lacking preserved siculae. **c-d**, **f**, specimens showing filiform nemae. **j**, fragmentary specimen showing tapering sicula continuous with nema. **g-i**, **k**, tubular fragments with visible zig-zagging and transverse sutures. **m**, **o-p**, large fragments of tubaria with uniseriate lateral thecae. Scale bars: 500 µm. Slide numbers and England Finder coordinates given in Data S1.

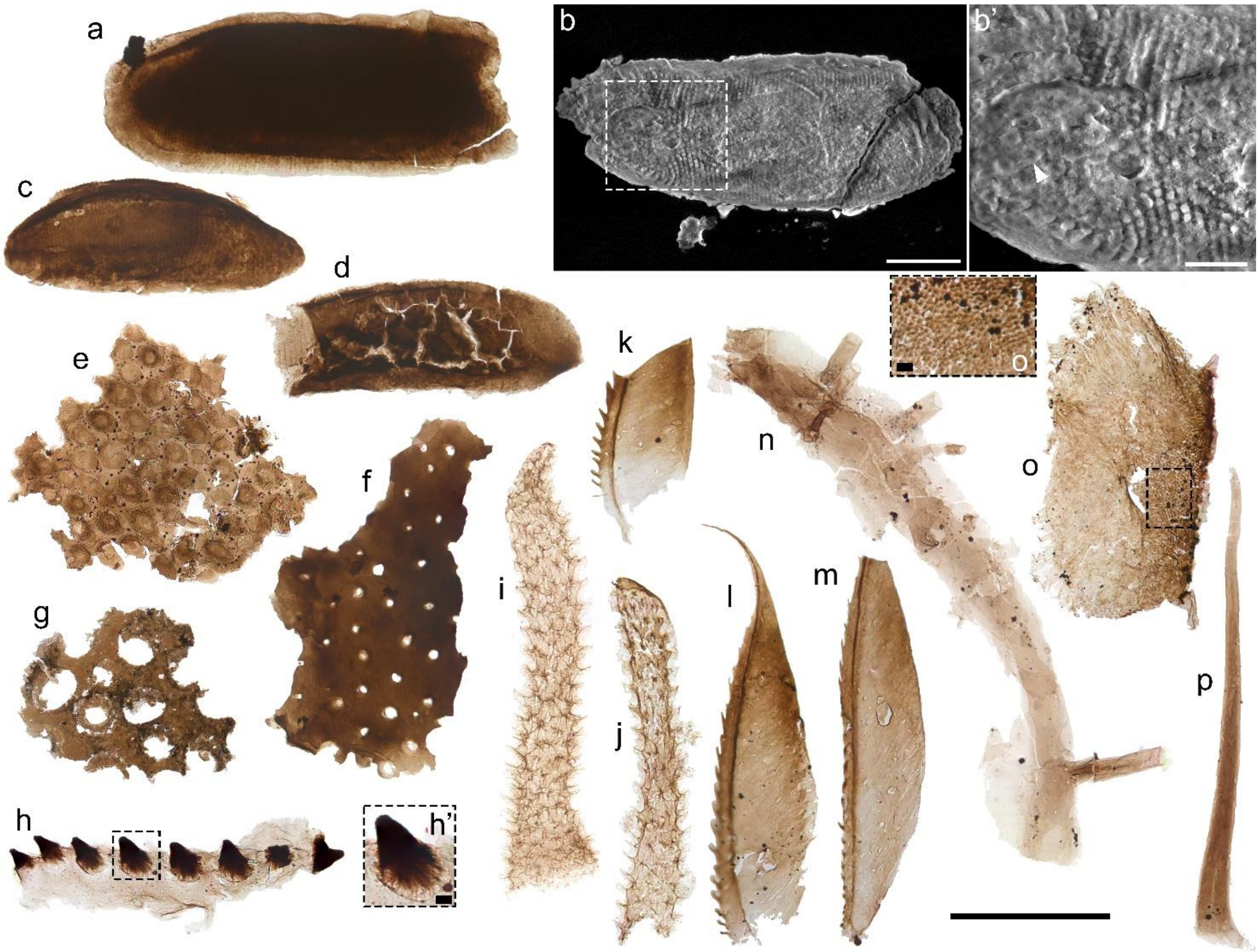

Fig. S5. Additional arthropod-type SCFs from the upper (a-d, f, i-j) and lower (e, g-h, k-p) Osterberg biota. a, c, d, molars photographed under optical microscopy. **b**, molar photographed under SEM, showing details of tubercles (b'). **e**, irregularly patterned tuberculate cuticle. **f**, finely perforated cuticle of indeterminate origin. **g**, cuticle with large rounded lacunae. **h**, uniseriate teeth from possible ramus of chelicera, with detail of tooth shown in h'. **i-j**, eurypterid-type respiratory organs. **k-m**, serrated cuticular 'paddles'. **n**, cuticle fragment with erect tubelike setae. **o**, cuticle with minute punctae, shown in o'. **p**, unornamented basally flaring spine. Scale bars: 100 μm except for b', h' (20 μm), a, c, d, h (200 μm), b (50 μm), n, o' (10 μm). Slide numbers and England Finder coordinates given in Data S1.

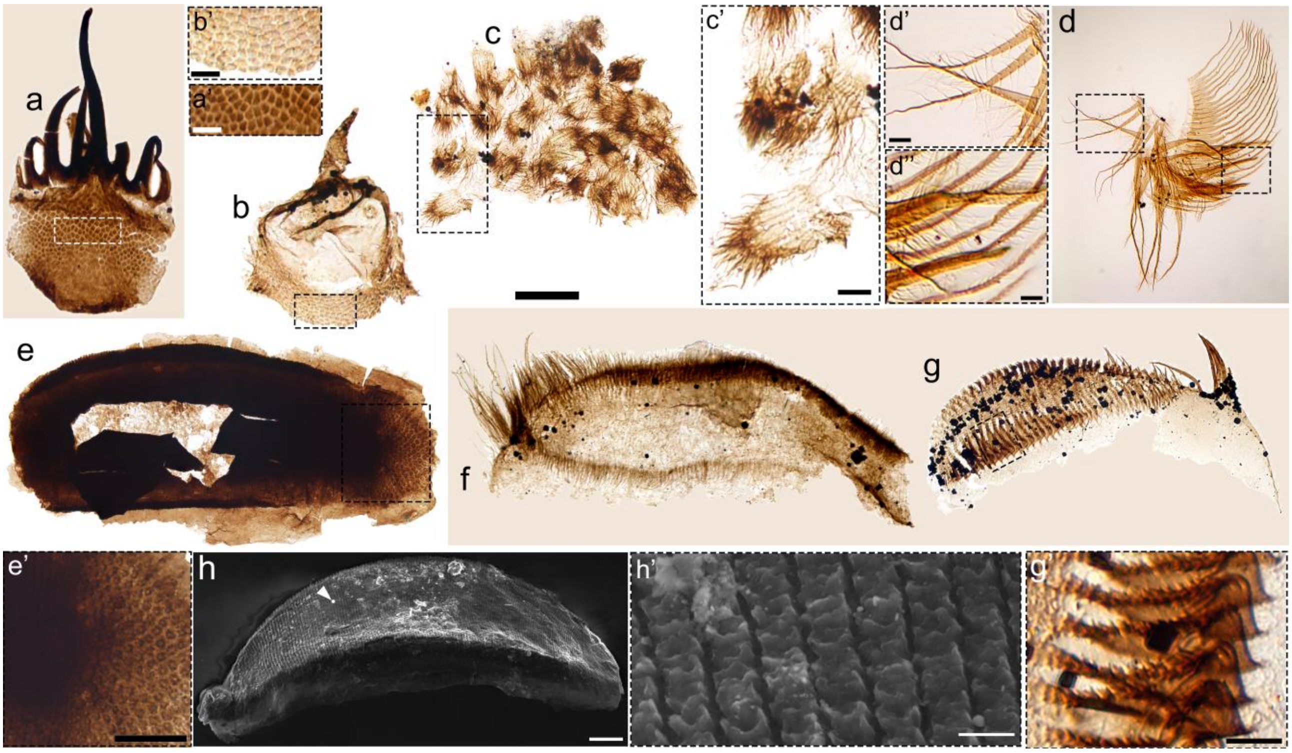

Fig. S6. Comparison of representative Osterberg SCFs (neutral background) with Cambrian specimens (beige background). **a**, ottoiid tooth from the middle/late Cambrian Deadwood Formation of Saskatchewan (31), with detail of scaly ornamentation shown in a'. **b**, priapulid sclerite from the lower Osterberg biota, with detail of scaly ornamentation shown in b'. **c**, filament-bearing priapulid sclerites from the lower Osterberg biota, with details of distal bristles shown in c'. **d**, crustaceomorph coplanar setose array from the middle/late Cambrian Deadwood Formation of Saskatchewan, with details of setules shown in d'-d''. **e**, crustaceomorph molar from the upper Osterberg biota, with detail of tuberculate ornamentation shown in e'. **f**, crustaceomorph molar from the early Cambrian Mount Clark biota. **g**, crustaceomorph molar from the middle/late Cambrian Deadwood Formation of Saskatchewan, with detail of grinding surface shown in g'. **h**, crustaceomorph molar from the upper Osterberg biota, with detail of grinding surface shown in h'. Scale bars: 50 µm except in a', b', c', d'-d'', h' (5 µm), e' (25 µm), e (100 µm). Slide numbers and England Finder coordinates given in Data S1.

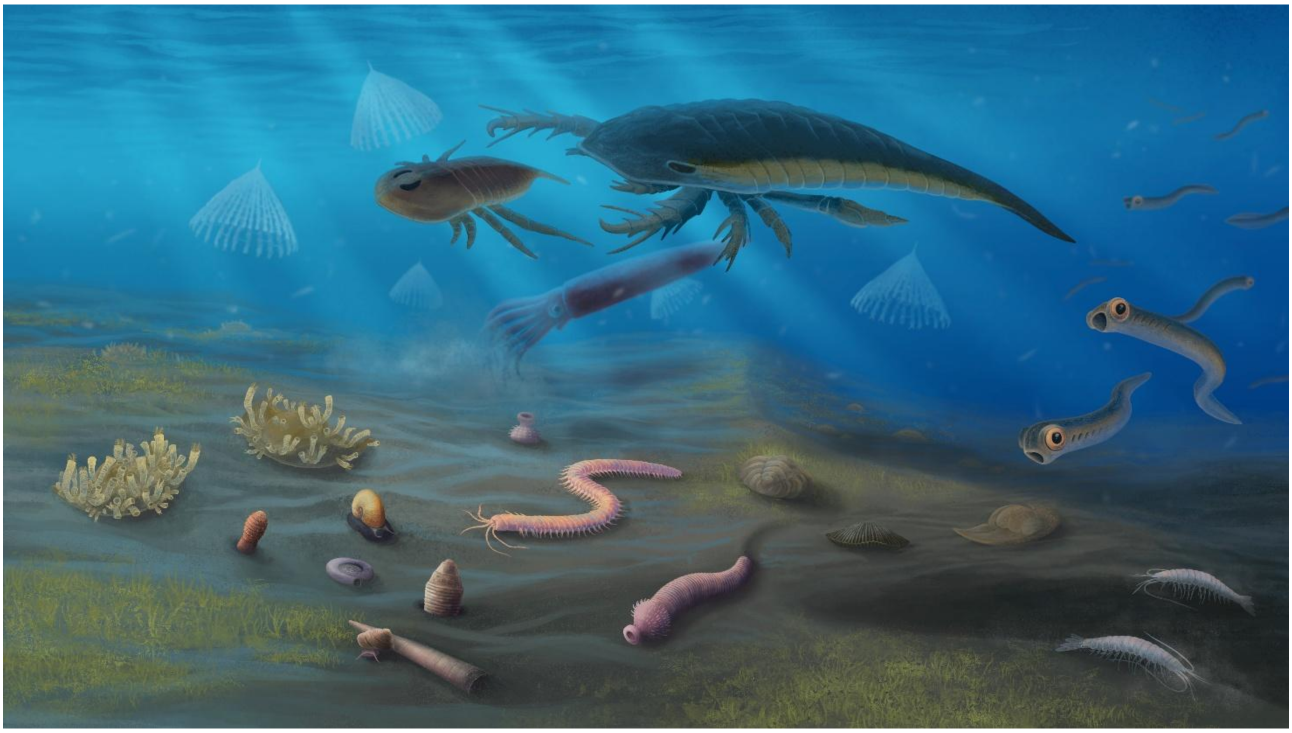

Fig. S7. **Artistic reconstruction of representative faunas from the lower (left) to upper (right) Osterberg biotas.** Reconstructed taxa are shown according to their approximate chronological order of appearance in the succession, from oldest (left; latest Cambrian-Tremadocian) to youngest (Darriwilian; right); distances are indicative only and do not reflect the exact stratigraphic horizons of appearance of taxa in the Osterberg drillcores.

Dataset S1 (separate file). Catalogue of SCFs from the Osterberg biotas indicating depths of provenance, slide numbers and England Finder coordinates.

SI References

1. S. Heredia, J. Carlorosi, A. Mestre, T. Soria, Stratigraphical distribution of the Ordovician conodont *Erraticodon* Dzik in Argentina. *J S Am Earth Sci* **45**, 224-234 (2013).
2. J. A. Bauer, Conodonts and conodont biostratigraphy of the Mclish and Tulip Creek formations (Middle Ordovician) of south-central Oklahoma. *Oklahoma Geological Survey Bulletin* **141**, 0078-4397 (1987).
3. J. Carlorosi, G. Sarmiento, S. Heredia, Selected Middle Ordovician key conodont species from the Santa Gertrudis Formation (Salta, Argentina): an approach to its biostratigraphical significance. *Geological Magazine* **155**, 878-892 (2018).
4. Y. Y. Zhen, J. Pickett, Ordovician (Early Darriwilian) conodonts and sponges from west of Parkes, central New South Wales. *Proceedings of the Linnean Society of New South Wales* **128**, 57-82 (2008).
5. M. Lindström, A suprageneric taxonomy of the conodonts. *Lethaia* **3**, 427-445 (1970).
6. S. Stouge, D. A. Harper, M. A. Parkes, Late Darriwilian (Middle Ordovician) conodonts from eastern and southeastern Ireland. *Irish Journal of Earth Sciences* **42**, 15-60 (2024).
7. S. Heredia, A. Mestre, The Middle Ordovician conodonts *Eoplacognathus robustus* Bergström and *E. lindstroemi* (Hamar): Taxonomy and apparatus reconstruction. *Andean Geol* **46**, 556-566 (2019).
8. D. L. Clark, J. F. Miller, Early evolution of conodonts. *Geological Society of America Bulletin* **80**, 125-134 (1969).
9. S. A. Leslie, S. M. Bergström, Element morphology and taxonomic relationships of the Ordovician conodonts Phragmodus primus Branson and Mehl, 1933, the type species of Phragmodus Branson and Mehl, 1933, and Phragmodus undatus Branson and Mehl, 1933. *J Paleontol* **69**, 967-974 (1995).
10. R. L. Ethington, D. L. Clark, Lower and Middle Ordovician Conodonts from the Ibex Area, Western Millard County, Utah. *Brigham Young University Geology Studies* **28**, 1-160 (1982).
11. Y. Y. Zhen, L. S. Normore, L. M. Dent, I. G. Percival, Middle Ordovician (Darriwilian) conodonts from the Goldwyer Formation of the Canning Basin, Western Australia. *Alcheringa* **44**, 25-55 (2020).
12. C. R. Barnes, D. J. Kennedy, A. D. McCracken, G. S. Nowlan, G. A. Tarrant, The structure and evolution of Ordovician conodont apparatuses. *Lethaia* **12**, 125-151 (1979).
13. M. Lindström, Conodonts from the lowermost Ordovician strata of south-central Sweden. *Geologiska Föreningen i Stockholm Förhandlingar* **76**, 517-604 (1954).
14. J. C. Lamsdell, D. E. G. Briggs, H. B. P. Liu, B. J. Witzke, R. M. Mckay, The oldest described eurypterid: a giant Middle Ordovician (Darriwilian) megalograptid from the Winneshiek Lagerstatte of Iowa. *Bmc Evolutionary Biology* **15** (2015).
15. H. Nowak, T. H. P. Harvey, H. B. P. Liu, R. M. McKay, T. Servais, Exceptionally preserved arthropodan microfossils from the Middle Ordovician Winneshiek Lagerstatte, Iowa, USA. *Lethaia* **51**, 267-276 (2018).
16. R. D. C. Bicknell, J. R. Paterson, J. B. Caron, C. B. Skovsted, The gnathobasic spine microstructure of recent and Silurian chelicerates and the Cambrian artiopodan Sidneyia: Functional and evolutionary implications. *Arthropod Struct Dev* **47**, 12-24 (2018).
17. S. Olive *et al.*, New insights into Late Devonian vertebrates and associated fauna from the Cuche Formation (Floresta Massif, Colombia). *J Vertebr Paleontol* **39** (2019).
18. G. Mussini, N. J. Butterfield, A microscopic Burgess Shale: small carbonaceous fossils from a deeper water biota and the distribution of Cambrian non-mineralized faunas. *Proceedings of the Royal Society B-Biological Sciences* **292** (2025).

19. D. T. De Moraes, S. G. B. C. Lopes, The functional morphology of *Neoteredo reynei* (Bartsch, 1920) (Bivalvia, Teredinidae). *J Mollus Stud* **69**, 311-318 (2003).
20. E. R. Lankester, J. T. Cunningham, "Mollusca" in Encyclopædia Britannica (11th ed.), H. Chisholm, Ed. (Hooper, Horace Everett, New York, USA, 1911), vol. 18, pp. 671.
21. J. B. Hou, N. C. Hughes, M. J. Hopkins, The trilobite upper limb branch is a well-developed gill. *Science Advances* **7** (2021).
22. M. R. Smith, T. H. P. Harvey, N. J. Butterfield, The macro- and microfossil record of the Cambrian priapulid *Ottoia*. *Palaeontology* **58**, 705-721 (2015).
23. S. Conway Morris, Fossil priapulid worms. *Special papers in Palaeontology* **20**, 1-95 (1977).
24. G. Mussini, N. J. Butterfield, Exotic cuticular specialisations in a Cambrian scalidophoran. *Proceedings of the Royal Society B* **292**, 20242806 (2025).
25. Y. H. Liu, S. H. Xiao, T. Q. Shao, J. Broce, H. Q. Zhang, The oldest known priapulid-like scalidophoran animal and its implications for the early evolution of cycloneuralians and ecdysozoans. *Evol Dev* **16**, 155-165 (2014).
26. G. Mussini, Y. P. Veenma, N. J. Butterfield, A peritidal Burgess-Shale-type fauna from the middle Cambrian of western Canada. *Palaeontology* **68**, e70001 (2025).
27. H. Q. Zhang *et al.*, Armored kinorhynch-like scalidophoran animals from the early Cambrian. *Sci Rep-Uk* **5** (2015).
28. B. J. Slater, T. H. P. Harvey, R. Guilbaud, N. J. Butterfield, A Cryptic Record of Burgess Shale-Type Diversity from the Early Cambrian of Baltica. *Palaeontology* **60**, 117-140 (2017).
29. N. J. Butterfield, & Harvey, T. H. P., Small carbonaceous fossils (SCFs): a new measure of early Paleozoic paleobiology. *Geology* **40**, 71-74 (2012).
30. T. H. P. Harvey, J. Ortega-Hernández, J. P. Lin, Y. L. Zhao, N. J. Butterfield, Burgess Shale-type microfossils from the middle Cambrian Kaili Formation, Guizhou Province, China. *Acta Palaeontol Pol* **57**, 423-436 (2012).
31. T. H. Harvey, Vélez, M. I., & Butterfield, N. J. (2012) Small carbonaceous fossils from the Earlie and Deadwood formations (middle Cambrian to lower Ordovician) of southern Saskatchewan. ed S. G. Survey (Saskatchewan Geological Survey), pp 1-8.
32. E. Wallet, B. Slater, S. Willman, J. S. Peel, Small Carbonaceous Fossils (SCFs) from North Greenland: new light on metazoan diversity in early Cambrian shelf environments. *Pap Palaeontol* **7**, 1403-1433 (2021).
33. E. Wallet, B. Slater, S. Willman, Organic-walled microfossils from the lower Cambrian of North Greenland: a reappraisal of diversity. *Palynology* **47** (2023).
34. B. Slater, S. Willman, Early Cambrian small carbonaceous fossils (SCFs) from an impact crater in western Finland. *Lethaia* **52**, 570-582 (2019).
35. N. J. Butterfield, R. H. Rainbird, Diverse organic-walled fossils, including "possible dinoflagellates," from the early Neoproterozoic of arctic Canada. *Geology* **26**, 963-966 (1998).
36. L. Y. Miao, Z. J. Yin, A. H. Knoll, Y. G. Qu, M. Y. Zhu, 1.63-billion-year-old multicellular eukaryotes from the Chuanlinggou Formation in North China. *Science Advances* **10** (2024).
37. L. L. Sloss, D. F. Merriam, Tectonic cycles of the North American craton. *Kansas Geological Survey Bulletin* **169**, 449-460 (1964).
